# Boldine modulates glial transcription and functional recovery in a murine model of contusion spinal cord injury

**DOI:** 10.3389/fncel.2023.1163436

**Published:** 2023-06-21

**Authors:** Carlos A. Toro, Kaitlin Johnson, Jens Hansen, Mustafa M. Siddiq, Walter Vásquez, Wei Zhao, Zachary A. Graham, Juan C. Sáez, Ravi Iyengar, Christopher P. Cardozo

**Affiliations:** ^1^Spinal Cord Damage Research Center, James J. Peters VA Medical Center, Bronx, NY, United States; ^2^Department of Medicine, Icahn School of Medicine at Mount Sinai, New York, NY, United States; ^3^Pharmacological Sciences, Icahn School of Medicine at Mount Sinai, New York, NY, United States; ^4^Institute for Systems Biomedicine, Icahn School of Medicine at Mount Sinai, New York, NY, United States; ^5^Departamento de Fisiología, Pontificia Universidad Católica de Chile, Santiago, Chile; ^6^Instituto de Neurociencias, Centro Interdisciplinario De Neurociencia De Valparaíso, Universidad de Valparaíso, Valparaíso, Chile; ^7^Florida Institute for Human and Machine Cognition, Pensacola, FL, United States; ^8^Department of Cell, Developmental, and Integrative Biology, University of Alabama, Birmingham, AL, United States; ^9^Research Service, Birmingham Veterans Affairs Health Care System, Birmingham, AL, United States; ^10^Rehabilitative Medicine, Icahn School of Medicine at Mount Sinai, New York, NY, United States

**Keywords:** boldine, contusion SCI, functional recovery, hemichannel blockade, spared white matter, glial response, connexins

## Abstract

Membrane channels such as those formed by connexins (Cx) and P2X_7_ receptors (P2X_7_R) are permeable to calcium ions and other small molecules such as adenosine triphosphate (ATP) and glutamate. Release of ATP and glutamate through these channels is a key mechanism driving tissue response to traumas such as spinal cord injury (SCI). Boldine, an alkaloid isolated from the Chilean boldo tree, blocks both Cx and Panx1 hemichannels (HCs). To test if boldine could improve function after SCI, boldine or vehicle was administered to treat mice with a moderate severity contusion-induced SCI. Boldine led to greater spared white matter and increased locomotor function as determined by the Basso Mouse Scale and horizontal ladder rung walk tests. Boldine treatment reduced immunostaining for markers of activated microglia (Iba1) and astrocytic (GFAP) markers while increasing that for axon growth and neuroplasticity (GAP-43). Cell culture studies demonstrated that boldine blocked glial HC, specifically Cx26 and Cx30, in cultured astrocytes and blocked calcium entry through activated P2X_7_R. RT-qPCR studies showed that boldine treatment reduced expression of the chemokine Ccl2, cytokine IL-6 and microglial gene CD68, while increasing expression of the neurotransmission genes Snap25 and Grin2b, and Gap-43. Bulk RNA sequencing revealed that boldine modulated a large number of genes involved in neurotransmission in spinal cord tissue just caudal from the lesion epicenter at 14 days after SCI. Numbers of genes regulated by boldine was much lower at 28 days after injury. These results indicate that boldine treatment ameliorates injury and spares tissue to increase locomotor function.

## Introduction

Spinal cord injury (SCI) is a devastating form of neurotrauma that results in life-long disabilities. However, with the exception of physical rehabilitation, there is no clinically available cell-based or pharmacologic approach to improve sensory or motor function after SCI (Badhiwala et al., 2018; Ahuja et al., 2020). The trauma results in a mechanical disruption of white and gray matter, shearing axons, crushing or shearing cell bodies, and causing damage or destruction of neural circuits (O’Shea et al., 2017). The mechanical injury initiates a series of tissue responses that further damage surviving cells through increased levels of reactive oxygen species (ROS), excitotoxic effects of glutamate and activation of glia (O’Shea et al., 2017). There then follows a cellular response that includes proliferation of astrocytes which surround the injury site, possibly to wall off the inflamed region (O’Shea et al., 2017). Sprouting of surviving neurons occurs, which results ultimately in formation of relay circuits that support partial recovery of function (Cafferty et al., 2010; Filipp et al., 2019).

A growing body of evidence links membrane channels such as hemichannels formes by connexins (Cx), or pannexins (Panx) and P2X_7_R channels to injury caused by tissue responses after SCI (Wang et al., 2004; Huang et al., 2012; Spray and Hanani, 2019; Abou-Mrad et al., 2020; Munoz et al., 2021). Under pathological conditions, these channels share several biophysical properties including permeability to ions. Cxs are a family of proteins that form hexameric pore structures called connexons, also referred to as Cx hemichannels (Cx HC), found largely in the cytoplasmic membrane but also in the membranes of organelles such as the mitochondria and endoplasmic reticulum. Individual Cxs proteins are named according to their molecular weights (Cisterna et al., 2014; Sáez et al., 2015) with connexin 43 (Cx43) is the most abundant Cx in the central nervous system where it is expressed in astrocytes, and to a lesser extent, in microglia (Giaume et al., 2021). Cx26 and Cx30 are also expressed on astrocytes of most species (Giaume et al., 2021). Cx HCs are best known as the building blocks of gap junctions (GJs) that form when Cx HCs on adjacent cells bind to one-another, forming a pore by which cells are electrically and chemically coupled. Cx HCs also exist as cell surface pores where they connect cytosol and extracellular fluids. Open Cx HC permit NAD^+^, adenosine triphosphate (ATP), and glutamate to leave the cell, raising their extracellular concentrations (Yi et al., 2017; Garcia-Rodriguez et al., 2022). An inward flow of calcium ions through open Cx HCs raises cytosolic calcium ion concentrations (Cisterna et al., 2014; Sáez et al., 2015; Giaume et al., 2021). Cx HCs are opened in response to various stimuli in physiological or pathological environments (Bennett et al., 2003) such as pro-inflammatory cytokines and oxidative and metabolic stress (Contreras et al., 2003; Retamal et al., 2007; Shintani-Ishida et al., 2007; Schalper et al., 2010). While under physiological conditions, open Cx HCs can modulate neuronal activity, in pathological situations they can also induce deterioration of cells and cell death (Evans et al., 2006; Chever et al., 2014). Pannexin 1 (Panx1) HCs are permeable to ATP and are present in astrocytes and in neurons in which they are mostly localized to the post-synaptic zone. P2X_7_R allow calcium ion to enter the cell when bound by ATP, are localized to microglia and are present in lesser amounts on astrocytes and pre-synaptic neurons (García-Rodríguez et al., 2022).

Previous studies of SCI in rodent models have demonstrated alterations in astrocytic Cx43 within the epicenter and penumbra of the lesion site (Theriault et al., 1997; Lee et al., 2005). The transformation of astrocytes into reactive astrocytes increases Cx43 expression as a direct result of traumatic SCI (Lee et al., 2005). Genetic ablation of Cx30 and Cx43 in astrocytes blunts ATP release and spares spinal cord tissue (Huang et al., 2012). Ependymal cell expression of Cx26 is developmentally regulated and is reduced in adults; spinal cord injury increases Cx26 expression in ependymal cells; levels of Cx26 HC decrease as new ependymal cells become coupled via GJs (Fabbiani et al., 2020).

It should be noted that extracellular ATP binds to and activates P2X_7_R purinergic receptors on microglia resulting in maturation and release of IL-1ß (Di Virgilio et al., 1999) and that P2X_7_R blockers reduced inflammation and improved functional recovery after SCI (Peng et al., 2009). In addition, binding of ATP to P2X_7_R channels adds to the inward calcium current and is linked to opening of Pnax1 HCs thereby further increasing release of ATP. Thus, P2X_7_R mediate a feed-forward amplification of signals originating as release of ATP through open Cx HCs through coupling of P2X_7_R with Panx1 (Suadicani et al., 2006).

It was recently discovered that boldine, a naturally occurring alkaloid extracted from the leaves and bark of the Chilean boldo tree (*Peumus boldus*), blocks Cx43 and Panx1 HC without affecting GJ communication (Yi et al., 2017). In a mouse model of Alzheimer’s disease, analysis of brain tissue showed that boldine prevented release of ATP and glutamate and lowered cytoplasmic calcium ion concentrations (Yi et al., 2017). These findings raised the question of whether boldine might reduce tissue injury and/or improve locomotor function after SCI. Here, we tested the hypothesis that administration of boldine beginning at 3 days after a moderate contusion SCI improves locomotor function and spare white matter.

## Materials and methods

### Animals

Use of live animals was conducted in accordance with PHS Policy on Humane Care and Use of Laboratory Animals and the Guide and was approved by the Institutional Animal Care and Use Committee at James J. Peters Veterans Affairs Medical Center (JJP VAMC) IACUC #CAR-20-11. Male and female C57Bl6 mice were purchased from Charles River and housed with controlled photoperiod (12/12 h light/dark cycle) and temperature (23–25°C) with *ad libitum* access to water and pelleted chow. Dark cycle was set from 6:00 AM to 6:00 PM. Animals were single housed one-week prior surgery. Experiments using cultured astrocytes were performed following protocols approved by Ethics Committee of the Universidad de Valparaíso, Chile; ID: #CICUAL F-03.

### Experimental design

**FIGURE 1:**
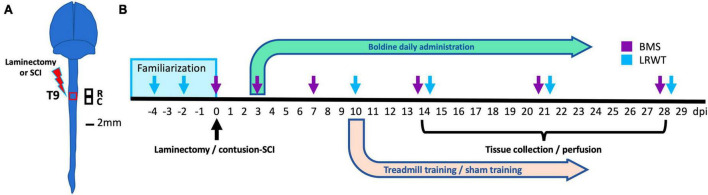
Experimental design. **(A)** Anatomical representation of the mouse brain and spinal cord showing the site of laminectomy or contusion SCI (red box) at the level of thoracic vertebrae 9 (T9). Black squares represent the locations of the rostral and caudal segments of spinal cord collected. These were used for histology, biochemistry and bulk RNA-seq studies. R: rostral; C: caudal. Scale bar, 2 mm. **(B)** Timeline for animal familiarization with handling, peanut butter and equipment, surgeries performed, boldine or vehicle administration, behavioral testing and timepoints for tissue collection and animal perfusion are depicted. BMS, Basso mouse scale; LRWT, ladder rung walk test.

A general summary of animal groups and sample sizes are provided in Table 1. At 4 months of age, male and female animals of similar weight (Supplementary Figures 1A, B) were randomly assigned to SCI or a sham-SCI. Each group was then randomly assigned to boldine or vehicle-treated groups (Table 1). The experimental design and timeline for the study procedures is shown in Figure 1.

**TABLE 1:** Experimental groups, procedures and treatments.

Surgery/ treatment	Timepoints	
	14-dpi	28-dpi
Laminectomy (sham)/Vehicle	*N* = 8 males; *N* = 6 females	*N* = 8 males; *N* = 6 females
Laminectomy (sham)/Boldine	*N* = 8 males; *N* = 6 females	*N* = 8 males; *N* = 6 females
Contusion SCI/Vehicle	*N* = 10 males; *N* = 8 females	*N* = 10 males; *N* = 8 females
Contusion SCI/Boldine	*N* = 10 males; *N* = 8 females	*N* = 10 males; *N* = 8 females
	Total number of animals = 128 (72 males and 56 females)	

### Spinal cord injuries

A motor-incomplete contusion SCI was performed using an Infinite Horizons (IH) impactor (Precision Systems and Instrumentation) as previously reported (Toro et al., 2021). Briefly, after induction of anesthesia by 3% isoflurane inhalation, animals were placed on heating pads warmed with recirculating water to 37°C. Hair was clipped over the cervical and thoracic spine areas. All animals underwent a laminectomy to expose the dura at level of thoracic vertebrae 9 (T9). The size of laminectomy was approximately 2 mm in diameter to allow the probe to impact the dura without touching any bone or other surrounding tissues. Randomized animals selected for the sham-SCI group had the vertebral muscle around the laminectomy site sutured to stabilize the spinal column, the incision site closed with 7 mm wound clips, and were returned to a clean cage on top of a warming pad. For animals selected for the SCI groups, they were placed on the clamping platform of the IH impactor under 3% isoflurane where their vertebral column was stabilized using the attached forceps to the IH clamping platform and received a 65 kdyne contusion SCI (Scheff et al., 2003). We chose this impact force as it results in a moderate-severe injury that allows for a partial recovery function over the timeframe of the study. Animals reach a maximal recovery and then plateau at around 28 days post-injury. After the lesion is generated, the left-right symmetry was confirmed by detection of equal bruising on both sides of the dorsal median sulcus. Actual impact force and cord displacement values were recorded for each animal and group (Supplementary Figure 2). Muscle was closed using resorbable sutures and skin closed with 7-mm wound clips. Post-operative care took place in cages with Alpha-Dri bedding (Newco Distributors, Inc., Hayward, CA, USA) over heating pads warmed with recirculating water for the first 72 h. All animals received pre-warmed lactated Ringers solution (LRS), carprofen and Baytril every 24 h for 5 d. Wound clips were removed at 10 days post-injury (dpi). Urine was expressed manually twice a day by gentle pressure and massage of the bladder until spontaneous voiding. Animals were fully checked for signs of stress, urine scalds, and autophagia at least once a day for the length of the study.

### Boldine administration

Daily boldine (Millipore Sigma, Jaffrey, NH, USA) administration started at 3 dpi. Boldine was added to cages at 2:00 PM. It was prepared by dissolving boldine in a mix of DMSO and peanut oil (Sigma). This mixture was then added to peanut butter (PB) so that 1.0 g of total bolus could be given once per day at a dose of 50 mg/kg. The final concentration of DMSO was less than 2.5%. Single-housed animals were familiarized with 1.0 gram of peanut butter for a week prior to surgery. All animals consumed 100% of their daily PB mix within 1 h and continued to do so throughout the remainder of the study. Vehicle-treated SCI and laminectomy-only animals (shams) received daily equal amount of the PB mix without boldine.

### Behavioral testing

Locomotor recovery after SCI was tested as previously reported (Toro et al., 2021) using the Basso mouse scale (BMS) open-field test (Basso et al., 2006), and the horizontal ladder rung walk test (LRWT) (Cummings et al., 2007), at specified timepoints (Figure 1). BMS is a well-established method for evaluating the severity of impairments in locomotor function after SCI by scoring locomotor milestones using a 9-point scale, with 0 being completely hindlimb paralysis and 9 being a normal healthy gait. The LRWT is used to evaluate fine motor skills and coordinated stepping as mice attempt to cross a commercially available horizontal ladder and were evaluated as they attempted to cross (Cummings et al., 2007). For BMS, two blinded investigators independently scored the animals and the results were averaged for a final score. For LRWT, animals were recorded with a video camera located under the ladder that was moved manually to keep the animal in-frame and later reviewed by two blinded observers who recorded the number of correct steps and errors. Animals were familiarized with equipment used for each of these behavioral tests one-week prior to surgery. We performed all behavior tests during the animals’ dark phase of the light:dark cycle, which was set from 6:00 AM to 6:00 PM for this study, as animals are normally more active in the dark. BMS and LRWT were performed between 9:30 AM and 11:30 AM.

### Tissue harvest

At days 14 and 28 post SCI, 5 animals per group (Table 1) were randomly selected to undergo perfusion-fixation under a deep anesthesia following an intraperitoneal injection of ketamine (100 mg/kg) and xylazine (30 mg/kg) as previously reported (Toro et al., 2021). Briefly, mice were euthanized by transcardial perfusion with sterile saline followed by injection if ice-cold 4% paraformaldehyde (PFA). After perfusion, spinal cords were removed and further post-fixed in 4% PFA for 72 h, transferred to a solution of 30% sucrose and stored at 4°C until cryostat sectioning. In addition, fresh spinal cord tissues from remaining animals of every group were collected after inducing deep anesthesia by inhalation of 3% isoflurane followed by decapitation. Spinal cord segments (∼2 mm) containing half the lesion epicenter and the region immediately rostral or caudal, were collected and snap frozen in liquid nitrogen and then stored at −80°C for biochemical analysis; tissues from comparable areas of spinal cords of sham-controls were also collected.

### Detection of white spared matter

Transverse 10-micron sections of perfusion-fixed spinal cords were obtained with a cryostat (Leica, West Hollywood, CA, USA) and used for immunofluorescence staining as previously reported (Toro et al., 2021). Myelin was stained using FluoroMyelin according to the manufacturer’s protocol (FluoroMyelin green, ThermoFisher, Carlsbad, CA, USA). Spinal cords sections from SCI animals from different groups were transversally cut rostral and caudal from the injury site every 100 microns for a total of 2 mm length centered on the injury epicenter. Sections were imaged with a confocal microscope (Carl Zeiss, Jena, Germany) as described below. To visualize differences in spared myelin an image analysis was performed using the ImageJ software as described elsewhere (Streijger et al., 2016). Briefly, thresholds for signals in the images were set at background gray values. The region of interest was determined and mean intensity and total number of pixels above threshold were measured. Spared tissue was determining by the percentage of white matter calculated by the total area of the spinal cord for each section using ImageJ.

### Immunofluorescence staining

Sections of perfusion-fixed spinal cord were used for immunofluorescence staining as previously reported (Toro et al., 2021). Antibodies were used to detect the following proteins: GAP-43 (Abcam #ab16053) to label regenerating axons and identify sprouting; GFAP (Abcam #ab7260) to detect reactive astrocytes; and Iba1(Abcam #ab178846) for labeling macrophages and activated microglia. Secondary antibodies, Alexa Fluor 488-conjugated goat anti-mouse IgG (Abcam # ab150113) and Alexa Fluor 647-conjugated goat anti-rabbit IgG (Abcam # ab150079). Details in Table 2.

**TABLE 2:** Antibodies for immunohistochemistry.

Antibody name	Isotype and host	Catalog number	Concentration	RRID
Anti-GAP43 antibody	IgG polyclonal, host rabbit	Abcam, ab16053	1:500	AB_443303
Anti-GFAP antibody	IgG polyclonal, host rabbit	Abcam, ab7260	1:500	AB_305808
Anti-Iba1antibody	IgG monoclonal, host mouse	Abcam, ab178846	1:500	AB_2636859
Alexa-488 anti-mouse	IgG polyclonal, host goat	Abcam, ab150113	1:2,500	AB_2576208
Alexa-647 anti-rabbit	IgG polyclonal, host goat	Abcam, ab150079	1:2,500	AB_2722623

### Fluorescent in-vitro mRNA hybridization

Fixed 10-micron spinal cord sections were mounted on Superfrost Plus slides (Thermo Fisher Scientific, Pleasanton, CA, USA). Customized probes for GFAP, Cx43 and S100a8 were designed and provided by Advance Cell Diagnostics (Hayward, CA, USA) for detection of mRNA. *In situ* hybridizations were performed according to the RNAscope Multiplex Fluorescent Reagent Kit v2 Assay protocol provided by the manufacturer and as described previously (Wang et al., 2012).

### Image capture and quantification

5 × 5 tiled images were obtained from stained sections using a 20× objective and a Zeiss 700 confocal microscope (Carl Zeiss, Jena, Germany). Blinded quantification was performed using ImageJ software (version 2.1.0/1.53c, National Institute of Health, USA) and integrated density of pixels was measured for each section and a mean value was calculated as previously described (Oliveira et al., 2004; Toro et al., 2021). Maximum background threshold was determined for each image and set for intensity quantification. Data are represented using the mean ± SEM.

### RNA extraction, reverse transcription and qPCR

Total RNA was extracted from spinal cord segments that extended either ∼2 mm rostral or ∼2 mm caudal to lesion epicenter using TRIzol reagent (ThermoFisher, Carlsbad, CA, USA) following the manufacturer’s instructions and methods previously described (Toro et al., 2018, 2021). Total RNA concentrations were determined by absorbance at 260 nm using a Nanodrop spectrophotometer (Thermo Scientific, Carlsbad, CA, USA). RNA was reverse-transcribed into cDNA using Omniscript reverse transcriptase (Qiagen, Germantown, MD, USA). PowerUp SYBR Green Master Mix (ThermoFisher, Carlsbad, CA, USA) was used to measure mRNAs of interest by qPCR. Primers were designed with help of the Primer Blast program from NCBI (Table 3). Formation of single SYBR Green-labeled PCR amplicons were verified by running melting curve analysis. Threshold cycles (CTs) for each PCR reaction were identified by using the QuantStudio 12K Flex software. To construct standard curves, serial dilutions were used from 1/2 to 1/512 of a pool of cDNAs generated by mixing equal amounts of cDNA from each sample. The CTs from each sample were referred to the relative standard curve to estimate the mRNA content per sample; the values obtained were then normalized for variations using peptidylprolyl isomerase A (Ppia) mRNA as the normalizing unit.

**TABLE 3:** Primer sequence for RT-qPCR.

Gene	Forward primer	Reverse primer	Product length (bp)	RefSeq
Ccl2	CTGGAGCATCCACGTGTTGG	TCCTTCTTGGGGTCAGCACAG	195	NM_011333.3
Ccl3	CCATGGGTCCCGTGTAGAGC	TGAAGAGTCCCTCGATGTGGC	113	NM_011337.2
Cd68	TCACCCGCAGACGACAATCA	AGATGAGGCGCTCCTTGGTG	85	NM_001291058.1
Cx43	TTCATTGGGGGAAAGGCGTGA	CACCCATGTCTGGGCACCTC	180	NM_010288.3
Cxcl1	CACTGCACCCAAACCGAAGTC	GGGAGCTTCAGGGTCAAGGC	72	NM_008176.3
Gap43	GGAGGAGCCTAAACAAGCCGA	CATCCTGTCGGGCACTTTCC	185	NM_008083.2
Gfap	AGAGAACAACCTGGCTGCGT	TGGCTTGGCCACATCCATCT	193	NM_001131020.1
Grin2b	GCAAGCCTGGCATGGTCTTC	CACGGATTGGCGCTCCTCTA	83	NM_001363750.1
IL-1β	TGCCACCTTTTGACAGTGATGA	TGCCTGCCTGAAGCTCTTGT	158	NM_008361.4
IL-6	TGATGGATGCTACCAAACTGGA	TGTGACTCCAGCTTATCTCTTGGT	197	NM_001314054.1
NefH	AGCTGCTCGGTCAGATCCAGG	CCTCTGAGAGTCGGTCCAACC	189	NM_010904.3
Ngf	AGGGGAGCGCATCGAGTTT	ACGCCGATCAAAAACGCAGT	197	NM_001112698.2
Mmp9	CTGGTGTGCCCTGGAACTCA	CACGTCGTCCACCTGGTTCA	140	NM_013599.4
Ppia	TGGTCAACCCCACCGTGTT	CCACCCTGGCACATGAATCCT	193	NM_008907.2
S100a8	ACTCGGACACTGAAGCCAGAG	CTCCAGCTCGGACATCCCG	73	NM_009115.3
Snap25	AGGCAAATGCTGTGGCCTTTT	TCTGCTCCCGTTCATCCACC	137	NM_001291056.1
Tnf	CAGGCGGTGCCTATGTCTCA	CAGCTGCTCCTCCACTTGGT	187	NM_001278601.1

### Transcriptomic profiling by RNA sequencing

We used total RNA extracted from each of two spinal cord segments that were collected from male mice at 14- and 28 days post SCI (*N* = 3 per timepoint), One of extended 2 mm rostral from the injury epicenter and the second 2 mm caudal from the injury epicenter. RNA integrity was checked using the RNA 6000 Nano assay (Agilent, Santa Clara, CA, USA). The sequencing library was prepared with a standard TruSeq RNA Sample Prep Kit v2 protocol (Illumina, San Diego, CA, USA), as described previously (Mariottini et al., 2019; Toro et al., 2021). RNA libraries were sequenced on the Illumina HiSeq 2000 System with 100 nucleotide pair end reads, according to the standard manufacturer’s protocol (Illumina, San Diego, CA, USA). For RNAseq data analysis, Star 2.5.4b and bowtie 2 2.1.0, samtools 0.1.7, Rsubread 2.10.5 and DESeq2 1.36.0 were used for read alignment to the mouse reference genome GRCm38.p6 using the ensemble gene annotation. At least 25 million reads were sequenced for each biological replicate (Supplementary Figure 3A). The percentage of reads that were successfully aligned to the mouse reference genome was between 84 and 89% (Supplementary Figure 3B). Differentially expressed genes (DEGs) were identified based on a maximum adjusted *p*-value of 10% (Supplementary Table 1). Up- and down-regulated gene sets were further interrogated using enrichR (Chen et al., 2013), with pathway enrichment analysis using Fisher’s exact test and the Gene Ontology Biological Processes 2018 library (Ashburner et al., 2000; Gene Ontology, 2021; Supplementary Table 2). Predicted pathways were ranked by significance. Significance *p*-values were transformed into -log_10_(*p*-values) and visualized as bar diagrams.

### Spinal cord astrocytes and HeLa cells to evaluate the activity of Cx26, Cx30, and P2X_7_R

Primary cultures of spinal cord astrocytes of 3 days old C57Bl6 mice were prepared using previously described methods (Kerstetter and Miller, 2012). Briefly, dissociated cells were resuspended in 10 ml DMEM with 10% FBS then transferred to a poly-lysine coated flask, place in an incubator at 37°C and 5% CO_2_, and allowed to adhere for 24 h. Flasks were then shaken at 200 rpm at 37°C overnight. The following day, non-adherent oligodendrocytes, neurons, and microglial were removed with aspiration and adherent cells were gently washed with DMEM containing 10% FBS, 10 mL of fresh DMEM with 10% FBS was added. Cells were allowed to recover and proliferate for 10 d before final seeding HeLa cells transfected with mouse Cx30 or Cx26 were used (kind donation from Christian Giaume, College de France, Paris, France and Klaus Willecke, Life and Medical Sciences Institute, Molecular Genetics, University of Bonn, Bonn, Germany, respectively). The activity of Cx HCs was evaluated using the dye uptake method described previously (Cea et al., 2013; Cisterna et al., 2020). In brief, cells were plated onto glass coverslips and bathed with Locke’s saline solution (all concentrations in mM: 154 NaCl, 5.4 KCl, 2.3 CaCl_2_, 1.5 MgCl_2_, 5 HEPES, 5 glucose, and pH 7.4) containing 5 μM DAPI, a molecule that crosses the plasma membrane through large-pore channels, including Cx HCs (Theriault et al., 1997). Since DAPI fluoresces upon its intercalation between DNA nucleotides, time-lapse recordings of fluorescent images were measured at regions of interest every 30 s for 13 min using a Nikon Eclipse Ti inverted microscope (Tokyo, Japan) and NIS-Elements software. The basal fluorescence signal was recorded in cells only in Locke’s saline solution that contained divalent cations. Then, cells were exposed to divalent cation-free solution (DCFS; Krebs buffer without CaCl_2_ and MgCl_2_), followed by 50 μM boldine and finally 200 μM La^3+^. Time-lapse fluorescence snapshot images were taken every 15 s. DAPI fluorescence was recorded in regions of interest using a Nikon Eclipse Ti microscope (Japan). Mouse P2X_7_R cDNA was cloned into pIRES-EGFP as previously described (Koshimizu et al., 1999) (kindly donated by Dr. Claudio Acuña, Instituto de Química y Biología, University of Santiago, Chile) and transiently transfected into HeLa cells to evaluate the activity of P2X_7_Rs. Intracellular calcium ion was detected using Fura-2AM, a ratiometric dye. Cells were incubated in Krebs-Ringer solution (all concentrations in mM: 145 NaCl, 5 KCl, 3 CaCl_2_, 1 MgCl_2_, 5.6 glucose, 10 HEPES-Na, pH7.4) containing FURA2-AM dye (2 μM) for 45 min at room temperature. The calcium signal was then measured using a Nikon Eclipse Ti microscope equipped with epifluorescence illumination, and images were obtained by using a Clara camera (Andor, Abingdon, UK) at 2 wavelengths of (λ) 340 nm and 380 nm, followed by calculating the ratio of fluorescence emission intensity after stimulation with each one of these two wavelengths. The activity of P2X_7_Rs was induced with 100 μM benzoyl ATP followed by the addition of 50 μM boldine. All measurements were performed in ∼20 cells per experiment in a total of at least four independent experiments.

### Statistical analysis

Statistical evaluations were performed with one-way or two-way mixed model analysis of variance (ANOVA), as indicated in the section “Results” and figure legends. *Post-hoc* comparisons were done using Tukey’s multiple comparison test. *P*-values of less than 0.05 were considered significant. Statistical calculations were performed using Prism 9 software (Graphpad, San Diego, CA, USA). Data are expressed as mean values are expressed as mean ± SEM.

## Results

### Boldine administration promotes functional recovery after a motor-incomplete SCI

**FIGURE 2:**
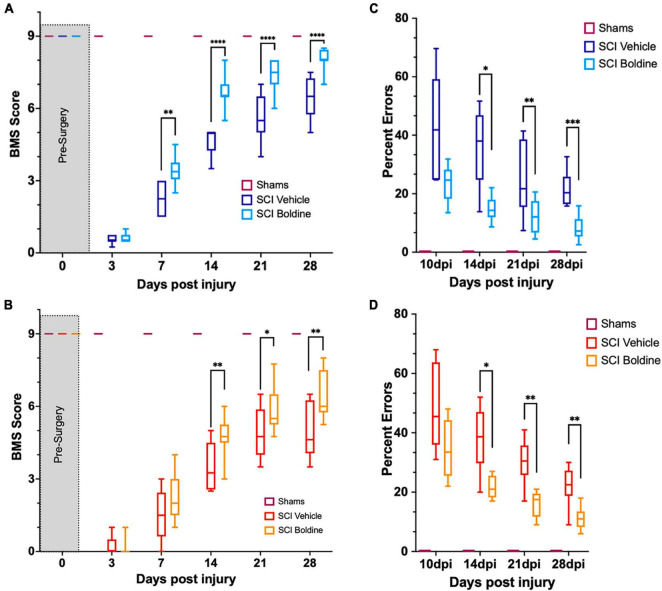
Boldine enhances functional recovery of C57BL/6 mice after contusion SCI. **(A)** BMS evaluation for shams, vehicle- and boldine treated male SCI mice; F(2, 27) = 891.1. **(B)** BMS evaluation for shams, vehicle- and boldine treated female SCI mice; F(2, 21) = 233.1. **(C)** LRWT scores for shams, vehicle- and boldine treated male mice; F(2, 27) = 136.3. **(D)** LRWT scores for shams, vehicle- and boldine treated female mice; F(2, 21) = 70.87. LRWT is expressed as percent of foot placement errors. Box-and-whisker diagrams represent the median, third quartile (upper edge) and first quartile (lower edge), and minimum and maximum values (whiskers) of the data. Statistical analysis was performed using a two-way mixed model ANOVA followed by a Bonferroni’s *post-hoc* test. *p < 0.05; ^**^p < 0.01; ^***^p < 0.001; ^****^p < 0.0001. *N* = 10 males; *N* = 8 females.

The effect of boldine on functional recovery after a thoracic motor-incomplete contusion SCI was evaluated by the open-field BMS test (Basso et al., 2006), and the horizontal LRWT (Cummings et al., 2007; Figure 2). Boldine or vehicle administration began at 3 dpi. All animals had maximum BMS scores before surgery (*p* > 0.999; Figures 2A, B). As expected, shams treated with either vehicle or boldine presented maximum BMS scores at all timepoints (Figures 2A, B). SCI animals treated with boldine or vehicle presented similar BMS scores at day 3 (0.62 vs. 0.61; *p* > 0.05 for males, and 0.14 vs. 0.25; *p* > 0.05 for females). However, scores for boldine-treated animals were significantly higher at 7 dpi in males when compared to SCI-vehicle mice by more than one-point on the BMS (3.41 vs. 2.29; *p* < 0.01). Boldine resulted in greater BMS mean scores in female mice compared to vehicle-treated animals at 7 dpi, though this did not reach our statistical threshold (2.25 vs. 1.53; *p* > 0.05). The differences between boldine- and vehicle-treated animals became greatest for both males and females at 14 dpi (6.56 vs. 4.61; *p* < 0.0001 for males, and 4.84 vs. 3.53; *p* < 0.01 for females) and stayed consistently elevated at 21 dpi (7.44 vs. 5.57 for males; *p* < 0.0001, and 5.89 vs. 4.88; *p* < 0.05 for females) and 28 dpi (7.94 vs. 6.11; *p* < 0.0001 for males, and 6.46 vs. 5.00: *p* < 0.01 for females) (Figures 2A, B). Omnibus effect for BMS was significant between treatment groups [F(2, 27) = 891.1 for males and F(2, 21) = 233.1 for females]. For LWRT, all sham animals, vehicle or boldine-treated, made no step placement errors (*p* > 0.999; Figures 2C, D). The SCI-vehicle group demonstrated a numerically higher percentage of foot placement errors when compared to the SCI-boldine group at 10 dpi (46.4 vs. 23.5% for males; *p* > 0.05, and 48.9 vs. 34.8%; *p* > 0.05 for females). These differences met statistical thresholds for significance for both sexes at 14 dpi (37.4 vs. 14.7%; *p* < 0.05 for males, and 38.0 vs. 21.6%; *p* < 0.05 for females), 21 dpi (30.2 vs. 12.0%; *p* < 0.01 for males, and 30.1 vs. 16.0%; *p* < 0.01 for females) and 28 dpi (23.3 vs. 8.9%; *p* < 0.001 for males, and 22.0 vs. 11.2%; *p* < 0.01 for females) (Figures 2C, D). Omnibus effect for LWRT was significant between treatment SCI-groups for both males [F(2, 27) = 136.3] and females [F(2, 21) = 70.87]. In addition, no significant sex differences were observed in vehicle-treated SCI animals by BMS [sex effect, *p* > 0.05, F(1, 16) = 4.277] or LRWT [sex effect, *p* > 0.05, F(1, 16) = 4.277]. However, we observed significant sex-differences between boldine-treated SCI animals by BMS [*p* < 0.001, F(1, 16) = 18, 26] and LRWT [*p* < 0.001, F(1, 16) = 9.090]. Altogether, our behavioral data suggests that boldine administration starting 3 d after a 65 kdyne contusion SCI induces significant improvement of gross and fine locomotor function in both male and female mice.

### Boldine administration after moderate severity contusion SCI increases spared white matter and reduces lesion volume

**FIGURE 3:**
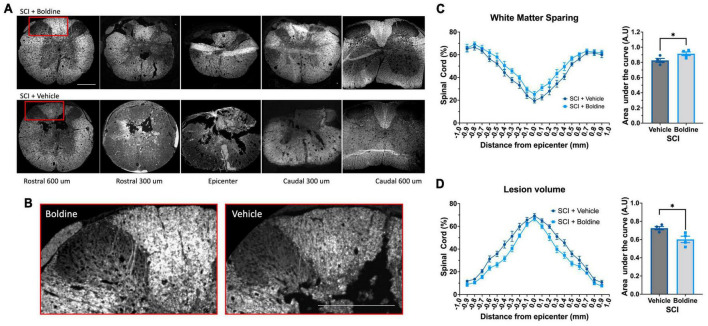
Boldine promotes sparing of white matter and reduces the lesion volume in male mice after SCI. Perfusion-fixed spinal cords of boldine and vehicle treated SCI animals were cryo-sectioned at 28 days. Transverse sections were collected every 100 μm and stained with FluoroMyelin. Panel **(A)** shows representative images at the epicenter and at 300 and 600 μm rostral and caudal from epicenter for each group. **(B)** Higher magnification of regions within the red boxes in panel **(A)** depicting differences between boldine and vehicle treated groups at 600 μm rostral from the epicenter. Scale bar is 500 μm. **(C,D)** Transverse sections stained with FluoroMyelin were analyzed every 100 μm at regions extending from 1 mm rostral to 1 mm caudal from the epicenter. **(C)** White matter sparing; F(2.477, 7.430) = 93.85; *p < 0.05 and **(D)** lesion site F(2.594, 7.782) = 202.2; *p < 0.05 were detected and compared between samples obtained from either boldine and vehicle-treated SCI groups. Area under the curve were calculated as detailed in the method section for each using ImageJ and Prism 9. Bar plots are presented as mean ± SEM. Statistical analysis was performed using unpaired *t*-test. *p < 0.05. *N* = 4 per group.

To determine whether the overall size of the lesion was affected by boldine administration at 28 days post SCI, we performed fluorescent myelin detection (FluoroMyelin; Thermo Scientific, Carlsbad, CA, USA) and confocal imaging of spinal cord transverse sections from vehicle or boldine-treated SCI males every 100 μm including the injury epicenter, and locations rostral and caudal to it (Figures 3A, B). Spared white matter was defined as the area stained with FluoroMyelin and the lesion size was measured as the disrupted area in each section. Area of spared white matter was calculated for each section after which an area under the curve was calculated and showed a significant increase in samples from boldine-treated SCI as compared to vehicle-treated SCI animals [F(2.477, 7.430) = 93.85; *p* < 0.05; Figure 3C]. Conversely, lesion volume was significantly reduced by boldine as compared to vehicle-treated SCI animals [F(2.594, 7.782) = 202.2; *p* < 0.05; Figure 3D]. Of note, samples collected at 14 dpi also showed an increase in white spared matter from SCI animals treated with boldine (Supplementary Figure 4). These data suggest that boldine reduces the lesion size and increase the abundance of white matter spared after SCI.

### Boldine reduces levels of reactive astrocytes and activated microglia and increases the expression of a protein involved in neuronal plasticity and growth cones after SCI

**FIGURE 4:**
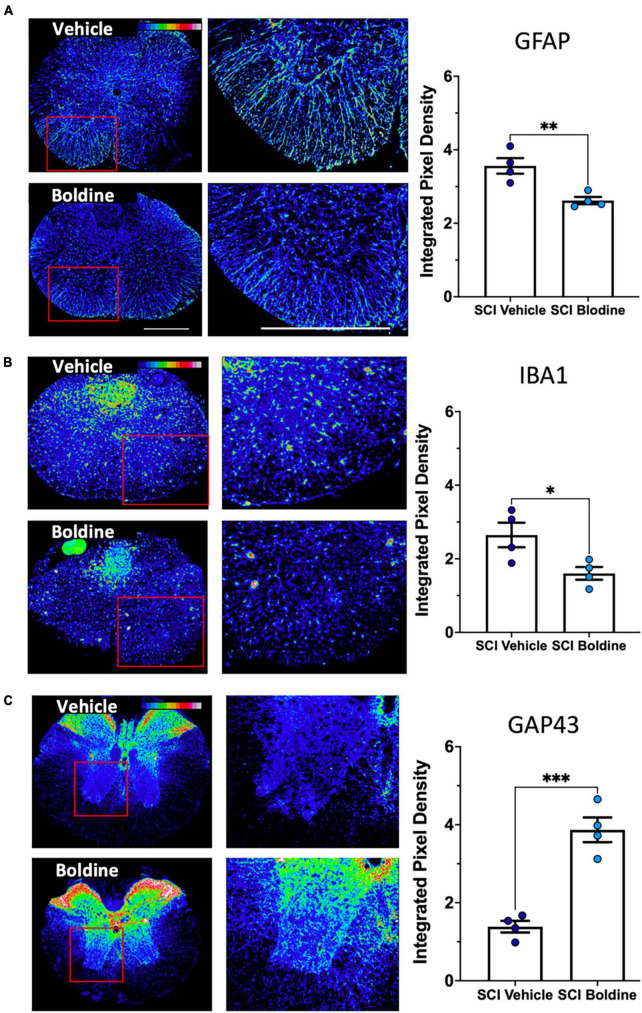
Boldine alters levels of protein markers for reactive astrocytes, activated microglia and neurite outgrowth after SCI. A total of 10 μm transverse spinal cord sections rostral to the lesion site collected at 14 dpi were immunostained and analyzed to detect changes between boldine and vehicle treated groups using: **(A)** reactive astrocyte marker GFAP; **(B)** activated macrophage / microglia marker Iba1; **(C)** axonal growth cone marker GAP43. Immunofluorescence intensity is depicted using a 16-colorized scale from black/blue (lowest) to red/white (highest) as shown in the top right corner of the first figure for each panel. Blinded quantification of immunolabeling, at the right of each panel, was performed by evaluating integrated pixel density using ImageJ and comparing the results between boldine and vehicle groups. Images displayed are representative examples from four SCI male mice, per group. Objective 20×. Scale bar, 500 microns. Bar plots show means ± SEM. Statistical analysis was done using unpaired *t*-test. ^***^p < 0.001, ^**^p < 0.005, *p < 0.01. *N* = 4.

We further compared the injured spinal cord of vehicle or boldine treated animals at 14 dpi by immunofluorescence (IF) examination. This timepoint was chosen because it reflects when locomotor recovery showed maximal differences between vehicle and boldine-treated groups. To assess the effect of boldine on gliosis after SCI, we probed sections taken just rostral to the lesion site for glial fibrillary acid protein (GFAP), a marker of reactive astrocytes (Brenner, 2014), and the ionized calcium binding adaptor molecule 1 (Iba1), a marker associated with macrophage and microglia activation (Peng et al., 2005; Miller et al., 2012). Fluorescent signals of both GFAP and Iba1 were significantly reduced by boldine (Figures 4A, B; *p* < 0.01 for GFAP and *p* < 0.05 for Iba1). Moreover, to investigate changes in expression of proteins related to neuronal plasticity and axon growth, we performed immunostaining of growth-associated protein 43 (GAP43) (Koshi et al., 2010). We showed that GAP43 fluorescence was significantly higher in sections rostral to the injury site of boldine-treated mice compared to vehicle-treated animals (Figure 4C; *p* < 0.001). These data suggest that boldine reduces glial reactivity and stimulates plasticity of spared fibers at 14 dpi after incomplete SCI. Of note, we did not detect significant effects in samples obtained at 28 dpi (data not shown).

### Boldine blocks Cx HC-mediated dye uptake in cultured embryonic spinal cord astrocytes

It is known that boldine blocks Cx43 and Panx1 HCs in the brain (Yi et al., 2017). To demonstrate that boldine blocks HCs present on the surface of spinal cord astrocytes, primary cultures of spinal cord astrocytes were maintained in divalent-cation-free culture media in the presence of DAPI, which is taken up by live cells through HCs (Yi et al., 2017), with or without 50 μM of boldine. Boldine significantly reduced uptake of DAPI during time-lapse imaging (Supplementary Figure 5; *p* < 0.001), indicating that it blocked open HCs in these cells.

### Boldine blocks Cx26 and Cx30 hemichannels and P2X_7_Rs

Multiple Cx other than Cx43 are expressed in the central nervous system, including Cx26 and Cx30 (Rash et al., 2001; Nagy et al., 2003). Therefore, we tested if boldine blocks dye uptake in open Cx26 and Cx30 HC using transfected HeLa cells. Dye uptake studies demonstrated that boldine slowed the rate of dye uptake through both Cx26 and Cx30 HCs (Supplementary Figure 6; *p* < 0.05). Addition of lanthanum ions, a well-stablished Cx HC blocker (Anselmi et al., 2008), did not further reduce the rate of dye uptake, confirming that boldine acted by blocking Cx26 and Cx30 HC. Because the effects of boldine on P2X_7_R uptake has not been tested, additional cytoplasmic calcium signal studies were performed. The data showed that 50 μM boldine also blocks P2X_7_R (Supplementary Figure 7).

### Boldine modulates spinal cord transcriptomic profiles after SCI

**FIGURE 5:**
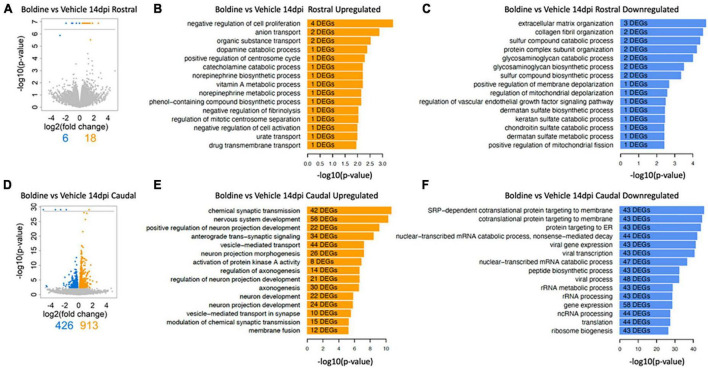
Boldine alters the injured spinal cord transcriptomic profile at 14 days after injury. Segments of spinal cord tissue collected at 14 dpi from mice with contusion SCI treated with either boldine or vehicle were subjected to bulk-RNA sequencing. Boldine-induced DEGs (FDR 10%) were identified between boldine and vehicle treated SCI animals either rostral **(A–C)** or caudal **(D–F)** from the injury epicenter. **(A,D)** Blue and orange dots indicate significantly up- or downregulated genes, respectively. DEGs predicted with a *p*-value of 0 and consequently -log_10_(*p*-value) of infinity are visualized above the horizontal line. **(B,C,E,F)** Up-regulated and downregulated genes for a particular condition were subjected to pathway enrichment analysis using Gene Ontology Biological Process and Fisher’s exact test, followed by ranking of the predicted pathways by significance. The top 15 ranked pathways are shown for all lists that describe differences between treatments rostral or caudal from the lesion site: **(B)** Boldine (vs. vehicle) rostral from the lesion upregulated, **(C)** boldine (vs. vehicle) rostral from the lesion downregulated, **(E)** boldine (vs. vehicle) caudal from the lesion upregulated and **(F)** boldine (vs. vehicle) caudal from the lesion downregulated. Numbers of DEGs observed in each particular pathway are shown within the bar for that pathway.

An unbiased understanding of the cellular and molecular mechanisms responsible for the differences observed in locomotor recovery between SCI animals treated with vehicle versus boldine was explored through bulk RNA-seq followed by a bioinformatic analysis. Total RNA was extracted from spinal cord segments rostral and caudal from the lesion epicenter (∼2 mm each) at 14 dpi, time when functional recovery differences were maximal in between treatments, and at 28 dpi, when functional recovery had reached a plateau in maximal locomotor function. Differentially expressed genes (DEG) were identified and the magnitude of change and number of DEGs that differed between boldine and vehicle treated SCI animals was reported (Figure 5 and Supplementary Figure 8). The data showed unique differences within the spinal cord segment at 14 dpi: while we found only 6 and 18 up- and downregulated genes in the spinal cord segment rostral from the lesion in boldine-treated animals (Figure 5A), we identified 426 and 913 up- and downregulated genes (Figure 5B) in the segment caudal from the lesion in boldine treated SCI mice, respectively.

To understand more in depth the biology associated with changes in numbers of DEGs, we identified biological processes represented by up- and downregulated genes rostral and caudal from the lesion site (Figures 5C–F). While the top 15 predicted pathways rostral from the lesion did not contain any pathways related to recovery of synaptic function or neuronal repair for any list of DEGs, the top upregulated pathways caudal from the lesion for boldine treated animals at 14 dpi focused on functions related to neuronal development, synaptic transmission and axonogenesis (Figure 5D). In contrast, the pathways that were downregulated caudal from the lesion included functions such as catabolic and biosynthetic processes (Figure 5F).

### Expression of pro-inflammatory molecules and genes involved in gliosis and neuronal function and plasticity are regulated by boldine after SCI

**FIGURE 6:**
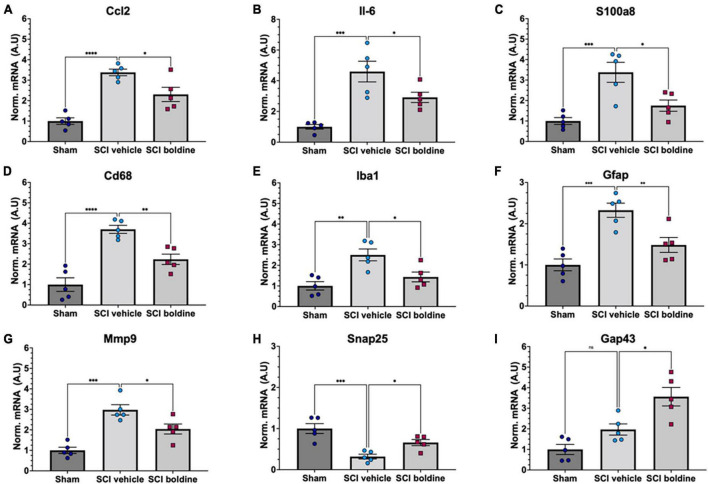
Effect of boldine in the modulation of mRNA levels of pro-inflammatory genes and genes related to gliosis, and neuronal plasticity and synaptic function after SCI. RT-qPCR was performed using total RNA isolated from 4 mm spinal cord segments spanning T7 to T11 (Figure 1) from sham and SCI mice at 14 dpi. Levels of **(A)** Ccl2; F(2, 8) = 20.33, **(B)** Il-6; F(2, 8) = 20.42, **(C)** S100a8; F(2, 8) = 11.12, **(D)** Cd68; F(2, 8) = 40.33, **(E)** Iba1; F(2, 8) = 10.01, **(F)** Gfap; F(2, 8) = 12.07, **(G)** Mmp9; F(2, 8) = 21.16, **(H)** Snap25; F(2, 8) = 12.56, and **(I)** Gap43; F(2, 8) = 18.29 were detected for laminectomy-only (Sham), and for SCI animals treated with vehicle (SCI vehicle) or boldine (SCI boldine). Data are expressed as arbitrary units (AU) after normalizing to SCI vehicle samples. Bar plots show mean ± SEM. Statistical analysis was performed by one-way ANOVA followed by Tukey’s multiple comparisons test *p < 0.05; ^**^p < 0.005; ^***^p < 0.001; ^****^p < 0.0001. *N* = 5.

To further investigate the effect of boldine in changes of mRNA levels after SCI, differences in targeted gene expression between vehicle and boldine treated animals were evaluated using spinal cord segments rostral and caudal from the lesion site at 14 dpi. RT-qPCR experimentation revealed significantly higher levels of pro-inflammatory molecules Ccl2 (Figure 6A; *p* < 0.0001), IL-6 (Figure 6B; *p* < 0.001) and S100a (Figure 6C; *p* < 0.001) in samples from SCI animals treated with vehicle as compared to shams. Interestingly, these changes were not detected in boldine-treated SCI samples, and were, in fact, significant between vehicle and boldine-treated SCI animals (Figures 6A–C; *p* < 0.05). Moreover, similar trends, although not significant, were observed for pro-inflammatory cytokines and chemokines Ccl3, Tnf, Cxcl1 and IL-1b (Brown et al., 2010), when comparing vehicle to boldine-treated SCI samples (Supplementary Figures 9A–D).

We then evaluated changes in the expression of Cd68 and Iba1, both makers of activated microglia (Xuan et al., 2019); Gfap, a highly expressed in reactive astrocytes (Brenner, 2014); and Mmp9 which encodes for an enzyme with degradation of extracellular matrix activity and protection of motor neuron death (Spiller et al., 2019). Levels of Cd68, Iba1, Gfap and Mmp9 were significantly increased in samples from vehicle-treated injured spinal cords in comparison to shams. However, these changes were not observed in samples from injured animals treated with boldine, and were significant when comparing vehicle-treated versus boldine-treated SCI groups (Figures 6D–G; *p* < 0.01 and *p* < 0.05). Levels of Cx43 also increased after injury (Supplementary Figure 9E; *p* < 0.0001), but they did not change when comparing sham to boldine-treated SCI samples (Supplementary Figure 9E; *p* > 0.05).

We also tested for genes that encode for SNAP25, involved in synaptogenesis (Antonucci et al., 2016), and Gap43 involved in axonal growth and plasticity (Koshi et al., 2010), and for the NMDA receptor subunit, Grin2b (Zhong et al., 1995). Interestingly, levels of Snap25 and Gap43 were significantly higher in samples from SCI animals treated with boldine as compared to vehicle-treated SCI samples (Figures 6G–I; *p* < 0.05). A similar trend was observed for Grin2b although not significant (Supplementary Figure 9F; *p* > 0.05). In addition, we evaluated changes for genes NefH and Ngf, also involved in axonal function, growth, maintenance and neuron survival (Ryden et al., 1997; Hayakawa et al., 2012; Turney et al., 2016; Fornaro et al., 2020) although changes between vehicle and boldine SCI samples were not significant and only showed a trend (Supplementary Figures 9G, H; *p* > 0.05).

**FIGURE 7:**
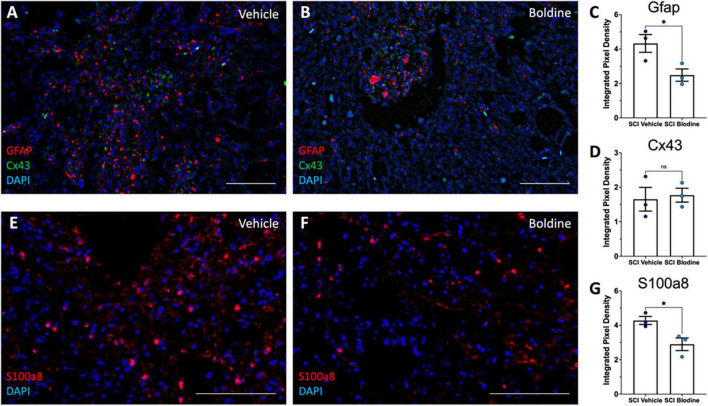
Boldine modulates mRNA levels of Gfap and S100a8. Transverse cryosections of spinal cords caudal to the lesion epicenter were collected at 14 dpi and processed for visualization of mRNA transcripts for Gfap (**A,B**; red), Cx43 (**A,B**; green) and S100a8 (**E,F**; red) using RNAScope. Samples were then visualized by confocal microscopy. Representative images show labeling of Gfap and Cx43 and S100a8 from vehicle treated animals **(A,E)** and boldine treated animals **(B,F)**. Staining intensity was quantified and plotted as integrated pixel density for Gfap (**C**; *p < 0.05), Cx43 (**D**; *p* = 0.7812) and S100a8 (**G**; *p < 0.05). Scale bar, 500 microns. Plots show mean value ± SEM. *, *p* < 0.05, unpaired 2-tailed *t*-test. *N* = 3.

We further performed RNAscope *in-situ* hybridization assay to detect mRNA transcripts and validate the changes observed for Gfap, S100a8, and Cx43 using transverse cryo-sections of spinal cords immediately caudal from the lesion epicenter of boldine and vehicle treated SCI animals at 14 dpi (Figure 7). Our results showed, as expected, significantly higher levels of Gfap and S100a8 in samples from vehicle-treated SCI animals as compared to boldine-treated SCI animals (Figures 7C, G; *p* < 0.05). However, levels of Cx43 transcripts remain similar at this timepoint (Figure 7D; *p* = 0.7812).

## Discussion

The above experiments tested if oral administration of boldine improved locomotor function after a moderate contusion SCI in mice. The major conclusion of this study is that boldine administration begun on day 3 after SCI resulted in increased locomotor function compared to vehicle-treated SCI animals as assessed by the BMS and LRWT. BMS test results were improved by as much as 2 points on this 9-point scale. Numbers of errors during LRWT were reduced by up to one half by boldine, suggesting substantial improvements in fine motor skills.

The present study was motivated by findings that boldine blocks Cx HCs (Yi et al., 2017) and by early work that showed the critical role of astrocytic Cx43 in the wave of ATP release that follows contusion SCI and which drives secondary injury through binding to and activation of P2X_7_R (Sluyter and Stokes, 2011; Huang et al., 2012). The importance of open Cx HCs in determining functional outcomes after contusion SCI was further demonstrated using monoclonal antibodies that block Cx HCs (Zhang et al., 2021). In those studies, the antibody reduced dye uptake within spinal cord at 4 h after SCI, increased locomotor function based on evaluation by BMS, increased spared tissue and spared neurons in perilesional regions of spinal cord and reduced astrogliosis (Zhang et al., 2021). Release of ATP through CxHC can activate a cascade of signaling that involves ligand-dependent activation of P2X_7_R and subsequent interactions between P2X_7_R and Cx HCs that increases Panx1 HC open probability further augmenting inward flow of calcium ions and outward movement of ATP. The finding that boldine blocks Panx1 HCs as well as P2X_7_Rs and Cx26 and Cx30 HCs suggest that boldine blocks multiple channels involved in this feed-forward inflammatory pathway.

Whether blockade of ATP release or P2X_7_R activation contributes to effects of boldine on functional recovery after SCI is unknown. ATP release may not be elevated by 3-days after SCI, when boldine administration was started, as the wave of ATP release that occurs in a rat model of contusion SCI was found to resolve by 24 h after SCI (Wang et al., 2004). Additionally, boldine has been shown to have multiple other pharmacological targets that include 5-HT_3_ receptors (Walstab et al., 2014), and endothelial selective small conductance calcium-activated K+ channels (de Souza et al., 2022). Further study is needed to determine the precise mechanisms by which boldine improves locomotor function after contusion SCI.

The experimental design incorporated a delayed start of boldine administration. One reason for this was that several prior studies established that blocking Cx HCs using the mimetic peptide 5 or inhibitory monoclonal antibodies improved locomotor function when treatment was begun soon after the contusion (O’Carroll et al., 2013; Mao et al., 2017; Zhang et al., 2021). It was not, however, known if substances that block Cx HCs could improve function when begun at later times after injury. The data reported here provide evidence that in mice with contusion SCI, boldine improved function even when its administration is delayed for 3 dpi. An unanswered question is whether boldine would further improve locomotor function after SCI if started at 1 h or 1 d after contusion. Studies showing that monoclonal antibodies administered intrathecally at 1 month after contusion did not improve locomotor function suggest that there is a time beyond which Cx HC blockers do not change the course of SCI (Zhang et al., 2021). Perhaps by this time, either all Cx HCs have closed or the cells expressing them are no longer situated near neurons and axons such that they are unable to affect health of these cells. More experimentation is needed to map out the temporal and spatial localization of open Cx HCs after SCI.

Spared white matter is a well-established determinant of locomotor function after contusion SCI (Basso et al., 1996). Findings that boldine treatment resulted in greater FluoroMyelin staining support white matter sparing as one mechanism by which boldine increased locomotor function. Interpretation of the effect of boldine on white matter should include some caveats. Specifically, BMS scores were indistinguishable at 3 dpi when comparing boldine-treated and vehicle-treated SCI groups but were higher in boldine-SCI mice compared to vehicle-SCI mice 4 days later. This suggests that initial damage to white matter was most likely comparable between boldine and vehicle groups and that boldine reduced subsequent white matter damage incurred beyond 3 days after the contusion. While it is well-accepted that demyelination and axon dieback continue for days to weeks after SCI (Alizadeh et al., 2019), effects of treatments or genetic modifications on loss of white matter beyond the first few minutes to hours after the SCI is a relatively understudied area of research. Our data provide experimental support for the possibility that glia responses to SCI could continue to stress axons days after injury and that the mechanisms can be inhibited by boldine.

The mechanisms for protection by boldine against delayed axon loss cannot be determined with certainty from our data. It is notable that immunostaining for the reactive astrocyte marker GFAP and the microglial marker Iga1 was increased in white matter in vehicle-SCI compared to boldine-SCI mice. It is quite likely that these cell types remain activated after SCI for days, contributing to release of reactive oxygen species, and pro-inflammatory cytokines. This interpretation is consistent with reduced activation of these cell types as assessed by immunofluorescence staining for protein markers, possibly lowering stresses on nearby axons. While logical, this interpretation requires experimental validation that should include evaluation of astrocyte and microglial responses to SCI across the entire lesion. Effects of boldine on expression of GFAP and Iba1 were determined at 14 dpi, when recovery of locomotor function is progressing and cellular and molecular processes that support such recovery are expected to be most evident. Further study is needed to determine temporal relationships of astrocyte and microglial changes with effects of boldine on functional outcomes.

Rewiring of the remaining neural circuitry is critical to functional recovery after SCI. The current understanding of such rewiring is that a key step requires formation relay circuits via axon branches arising from axons rostral from the site of axon injury; these branches project to regions in the brain or spinal cord where they synapse with cell bodies of neurons that project axons through spared white matter to cell bodies caudal from the lesion (Bareyre et al., 2004; Courtine et al., 2008; Asboth et al., 2018). Here, additional axon branches may be formed to synapse with alpha motor neurons either directly or through interneurons (Asboth et al., 2018). Some of the relay circuits formed in this way have been experimentally shown be responsible for recovery of locomotor function (Asboth et al., 2018). Formation of these relay circuits must require specific gene expression programs to support axon growth and remodeling of extracellular matrix. Our data do not permit us to determine whether boldine increased formation of relay circuits. However, the data do provide indirect evidence that boldine increased remodeling of local neural circuitry. Specifically, spinal cords of boldine-SCI mice demonstrated greater immunolabeling for protein markers of axon growth cones, and higher levels of mRNA for synaptic function. However, these data must be interpreted with caution for several reasons. The bulk-RNAseq data did not reveal alterations in genes involved in axon growth; it is possible such changes occurred but did not pass the criteria used for filtering out genes, or because the timeframe for peak expression of these genes was much earlier. It is possible that GAP43 is upregulated in non-neuronal cells which would confound interpretation of the findings. Further studies using tract tracing and synaptic silencing are needed to understand whether boldine indeed alters formation of relay circuits.

A surprising finding was that bulk RNA sequencing showed few DEGs in tissue samples just rostral from the lesion but many DEG caudal from it; DEG observed in spinal cord tissue just caudal from the lesion represented multiple gene ontologies involved in neurotransmission. One interpretation of these findings is that because axons are tiny relative to cell bodies, the changes in mRNA levels responsible for increased axonal expression of GAP-43 are not detected against the overall levels and magnitude of changes of mRNAs from whole tissue homogenate. In contrast, caudal from the lesion, there may be many neurons responding to new synaptic inputs received from new axon branches eliciting large changes in gene expression programs in these cell bodies. This interpretation is consistent with findings from recent studies from the Levine lab have shown that extensive changes in expression of genes occur in selected populations of neurons in lumbar spinal cord after a severe thoracic contusion SCI (Russ et al., 2021); Further study is needed to define the impact of boldine on neurons caudal from the lesion after SCI.

There are two limitations of our bulk RNAseq data. Bulk RNAseq does not allow us to identify the cell type(s) in which gene expression changes occur. Thus, we cannot exclude the possibility that some biologically important gene expression changes were not detected because upregulation in one cell type was canceled out by downregulation in another.

Boldine appeared to improve BMS scores more in male than female mice. Reasons for these sex-specific effects are unknown. The estrous cycle of female mice was not synchronized which could in theory explain any differences in variability of behavioral outcomes between sexes but seems less likely as the cause of the smaller effect of boldine on locomotor function of females after SCI. While there are no reports of interactions between boldine and steroid hormone action it remains possible that boldine alters signaling via estrogen or androgen receptors or the rapid, steroid hormone receptor-independent, transient signaling elicited by steroid hormones at the cytoplasmic membrane of various cells including neurons (Estrada et al., 2006). Our recent analysis of the effects of boldine on the multiome of skeletal muscle at 7 or 28 days after spinal cord transection identified androgen signaling as being impacted in muscle by boldine (Potter et al., 2023). Further investigation is needed to understand the biological basis for the greater effects of boldine on locomotor function in males after spinal cord contusion.

Following SCI, astrocytes promote tissue injury (Huang et al., 2012), contribute to a glial scar at the boarder of the contusion and facilitate functional recovery after SCI (Okada et al., 2018). An interesting effect of boldine treatment was to reduce intensity of GFAP staining suggesting that boldine reduced either mass of astrocytes, numbers of reactive astrocytes expressing increased GFAP or both. Our interpretation of the findings is that by blocking astrocytic Cx HCs the signals that activate astrocytes after SCI through entry of calcium and/or release of glial transmitters such as glutamate and ATP is reduced. Whether these effects are beneficial or deleterious cannot be determined from our data.

In conclusion, the findings of this study show that boldine spares white matter and improves locomotor function in mice with a moderate severity mid-thoracic spinal cord contusion. Limitations include an incomplete understanding of molecular mechanism by which boldine achieves these effects and uncertainty regarding effects of time after SCI at which boldine is administered on its ability to improve locomotor function after SCI. It will be exciting to learn results of experiments addressing these limitations.

## Data availability statement

The data presented in this study are deposited in the GEO repository, accession number GSE220907.

## Ethics statement

The animal study was reviewed and approved by the Institutional Animal Care and Use Committee at James J. Peters Veterans Affairs Medical Center (JJP VAMC) IACUC #CAR-20-11.

## Author contributions

CC, JS, and CT: conceptualization. CT, KJ, MS, WV, JS, and JH: methodology. JH and RI: software. CT, WZ, ZG, MS, WV, and CC: investigation. CT, JH, KJ, WV, and MS: formal analysis. CC, JS, and RI: resources and supervision. CT and CC: writing—original draft. CT, WZ, MS, JH, ZG, JS, RI, JS, and CC: writing—review and editing. CT, MS, JH, and JS: visualization. CC and RI: funding acquisition. All authors contributed to the article and approved the submitted version.

## Supplementary material

Supplementary Figure 1 Body weights were determined on the day of surgery prior to making an incision (pre-op) and at the specified times shown on the *X*-axis for male **(A)** and female **(B)** mice. Data are shown as mean values ± SEM.

Supplementary Figure 2 Actual impact force (kdyne) **(A)** and spinal cord displacement during impact (μm) **(B)** are shown. Data are shown as mean ± SEM.

Supplementary Figure 3 Transcriptomic profiling by RNA sequencing. “Star” was used for read alignment to the mouse reference genome. **(A)** Read counts and (# Reads) **(B)** alignment efficiencies are shown for each sample.

Supplementary Figure 4 Boldine promotes sparing of white matter at 14 days post SCI. Perfusion-fixed spinal cords of boldine and vehicle treated SCI animals were cryo-sectioned. Transverse sections were collected at 100 and 300 μm rostral and caudal from the injury the epicenter and stained with FluoroMyelin. Panel **(A)** shows representative images at +300, +100, −100 and −300 μm for each group. **(B)** White matter sparing was compared between boldine and vehicle-treated SCI groups. Bar plots are presented as mean ± SEM. Statistical analysis was performed using unpaired *t*-test. ^**^*p* < 0.01 *N* = 3 per group. Scale bar is 500 μm.

Supplementary Figure 5 Boldine blocks hemichannels expressed by activated spinal cord astrocytes. Spinal cord astrocytes were cultured under control conditions or treated with 10 ng/ml TNF-α plus 10 ng/ml IL-1β 1 at different time periods and dye uptake measured as fluorescence intensity in arbitrary units (AU) was evaluated under basal conditions, after exposure to extracellular divalent cation-free solution (DCFS) followed by the application of 50 μM boldine at the times denoted by the arrows in **(A)** and **(B)**. Curve of fluorescence intensity over time for dye uptake rate was calculated for each condition **(C)**. Each value represents the mean ± SEM of three independent experiments. A total of 30 cells were recorded per experiment.

Supplementary Figure 6 Boldine blocks Cx26 and Cx30 hemichannels. **(A,B)** Cx HC activity was assessed by DAPI uptake measure in time-lapse in Krebs solution, in DCFS to increase the open probability of hemichannels, and in DCFS plus 50 μM boldine. **(C)** DAPI uptake rate in HeLa Cx26 y HeLa Cx30, the application of DCFS increases the slope of the dye uptake curve and the application boldine in DCFS drastically reduced the dye uptake. *N* = 4, 30 cells were recorded for each experiment, values are presented as the mean ± SEM. **p* < 0.05. Tukey’s multiple comparisons test.

Supplementary Figure 7 Boldine blocks P2X_7_R. The activity of P2X_7_R was evaluated in HeLa cells transiently transfected with mouse P2X_7_R -EGFP. Cells were loaded with Fura-2 and intracellular calcium signal was evaluated. Upon treatment with 100 μM benzoyl ATP (inset in the left panel) a calcium signal increase was evident. In some experiments 50 μM boldine (arrows) was added simultaneously with BzATP (left panel) or after cells were treated with BzATP (right panel). Each plotted point corresponds to the mean value of 20 cells of a representative experiment out of four independent experiments.

Supplementary Figure 8 Results of analysis by bulk-RNA sequencing of total RNA isolated from spinal cord segments rostral and caudal from the lesion site at 28 dpi. Spinal cord segments collected at 28 dpi from mice with contusion SCI treated with boldine or vehicle were subjected to bulk-RNA sequencing. Boldine-induced DEGs (FDR 10%) were identified between boldine and vehicle treated SCI animals either rostral **(A–C)** or caudal **(D–F)** from the injury epicenter. **(A,D)** Blue and orange dots indicate significantly up- or downregulated genes, respectively. DEGs predicted with a *p*-value of 0 and consequently −log_10_(*p*-value) of infinity are visualized above the horizontal line. **(B,C,E,F)** Up-regulated and downregulated genes for a particular condition were subjected to pathway enrichment analysis using Gene Ontology Biological Process and Fisher’s Exact test, followed by ranking of the predicted pathways by significance. The top 15 ranked pathways are shown for all lists that describe differences between treatments rostral or caudal from the lesion site: **(B)** Boldine (vs. vehicle) rostral from the lesion upregulated, **(C)** Boldine (vs. vehicle) rostral from the lesion downregulated, **(E)** Boldine (vs. vehicle) caudal from the lesion upregulated, and **(F)** Boldine (vs. vehicle) caudal from the lesion downregulated. Numbers of DEGs observed in each particular pathway are shown within the bar for that pathway. Procedures were as described in the Section “Materials and methods” and the legend to Figure 5.

Supplementary Figure 9 Additional RT-qPCR Results. RT-qPCR was performed using total RNA isolated from 4 mm spinal cord segments spanning T7 to T11 from sham and SCI mice at 14 dpi. Levels of **(A)** Ccl3; F(2, 8) = 15.62, **(B)** Tnf; F(2, 8) = 4.54, **(C)** Cxcl1 F(2, 8) = 10.23, **(D)** Il-1b; F(2, 8) = 13.88, **(E)** Cx43; F(2, 8) = 36.82, **(F)** Grin2b; F(2, 8) = 5.727, **(G)** Nefh; F(2, 8) = 13.37, and **(H)** Ngf; F(2, 8) = 2.16 were detected for laminectomy-only (Sham), and for SCI animals treated with vehicle (SCI vehicle) or boldine (SCI boldine). Data are expressed as arbitrary units (AU) after normalizing to SCI vehicle samples. Bar plots show mean ± SEM. Statistical analysis was performed by one-way ANOVA followed by Tukey’s multiple comparisons test **p* < 0.05; ^**^*p* < 0.005; ^***^*p* < 0.001; ^****^*p* < 0.0001. *N* = 5.

Supplementary Table 1 Differentially expressed genes between boldine vs. vehicle rostral and caudal from the lesion site at 14 and 28 dpi.

Supplementary Table 2 Predicted top 15 Gene Ontology Biological Processes for each list of differentially expressed genes.

## References

[B1] Abou-Mrad Z. Alomari S. Bsat S. Moussalem C. Alok K. El Houshiemy M. (2020). Role of connexins in spinal cord injury: an update. *Clin. Neurol. Neurosurg.* 197:106102. 10.1016/j.clineuro.2020.106102 32717564

[B2] Ahuja C. S. Mothe A. Khazaei M. Badhiwala J. Gilbert E. van der Kooy D. (2020). The leading edge: emerging neuroprotective and neuroregenerative cell-based therapies for spinal cord injury. *Stem Cells Transl. Med.* 9 1509–1530. 10.1002/sctm.19-0135 32691994 PMC7695641

[B3] Alizadeh A. Dyck S. M. Karimi-Abdolrezaee S. (2019). Traumatic spinal cord injury: an overview of pathophysiology, models and acute injury mechanisms. *Front. Neurol.* 10:282. 10.3389/fneur.2019.00282 30967837 PMC6439316

[B4] Anselmi F. Hernandez V. Crispino G. Seydel A. Ortolano S. Roper S. (2008). ATP release through connexin hemichannels and gap junction transfer of second messengers propagate Ca2+ signals across the inner ear. *Proc. Natl. Acad. Sci. U S A.* 105 18770–18775. 10.1073/pnas.0800793105 19047635 PMC2596208

[B5] Antonucci F. Corradini I. Fossati G. Tomasoni R. Menna E. Matteoli M. (2016). SNAP-25, a known presynaptic protein with emerging postsynaptic functions. *Front. Synaptic Neurosci.* 8:7. 10.3389/fnsyn.2016.00007 27047369 PMC4805587

[B6] Asboth L. Friedli L. Beauparlant J. Martinez-Gonzalez C. Anil S. Rey E. (2018). Cortico-reticulo-spinal circuit reorganization enables functional recovery after severe spinal cord contusion. *Nat. Neurosci.* 21 576–588. 10.1038/s41593-018-0093-5 29556028

[B7] Ashburner M. Ball C. Blake J. Botstein D. Butler H. Cherry J. (2000). Gene ontology: tool for the unification of biology. the gene ontology consortium. *Nat. Genet.* 25 25–29. 10.1038/75556 10802651 PMC3037419

[B8] Badhiwala J. H. Ahuja C. S. Fehlings M. G. (2018). Time is spine: a review of translational advances in spinal cord injury. *J. Neurosurg. Spine* 30 1–18. 10.3171/2018.9.SPINE18682 30611186

[B9] Bareyre F. M. Kerschensteiner M. Raineteau O. Mettenleiter T. Weinmann O. Schwab M. (2004). The injured spinal cord spontaneously forms a new intraspinal circuit in adult rats. *Nat. Neurosci.* 7 269–277. 10.1038/nn1195 14966523

[B10] Basso D. M. Beattie M. S. Bresnahan J. C. (1996). Graded histological and locomotor outcomes after spinal cord contusion using the NYU weight-drop device versus transection. *Exp. Neurol.* 139 244–256. 10.1006/exnr.1996.0098 8654527

[B11] Basso D. M. Fisher L. Anderson A. Jakeman L. McTigue D. Popovich P. (2006). Basso mouse scale for locomotion detects differences in recovery after spinal cord injury in five common mouse strains. *J. Neurotrauma* 23 635–659. 10.1089/neu.2006.23.635 16689667

[B12] Bennett M. V. Contreras J. Bukauskas F. Sáez J. (2003). New roles for astrocytes: gap junction hemichannels have something to communicate. *Trends Neurosci.* 26 610–617. 10.1016/j.tins.2003.09.008 14585601 PMC3694339

[B13] Brenner M. (2014). Role of GFAP in CNS injuries. *Neurosci. Lett.* 565 7–13. 10.1016/j.neulet.2014.01.055 24508671 PMC4049287

[B14] Brown C. M. Mulcahey T. Filipek N. Wise P. (2010). Production of proinflammatory cytokines and chemokines during neuroinflammation: novel roles for estrogen receptors alpha and beta. *Endocrinology* 151 4916–4925. 10.1210/en.2010-0371 20685874 PMC2946152

[B15] Cafferty W. B. Duffy P. Huebner E. Strittmatter S. (2010). MAG and OMgp synergize with Nogo-A to restrict axonal growth and neurological recovery after spinal cord trauma. *J. Neurosci.* 30 6825–6837. 10.1523/JNEUROSCI.6239-09.2010 20484625 PMC2883258

[B16] Cea L. A. Cisterna B. Puebla C. Frank M. Figueroa X. Cardozo C. (2013). De novo expression of connexin hemichannels in denervated fast skeletal muscles leads to atrophy. *Proc. Natl. Acad. Sci. U S A.* 110 16229–16234. 10.1073/pnas.1312331110 24043768 PMC3791696

[B17] Chen E. Y. Tan C. Kou Y. Duan Q. Wang Z. Meirelles G. (2013). Enrichr: interactive and collaborative HTML5 gene list enrichment analysis tool. *BMC Bioinformatics* 14:128. 10.1186/1471-2105-14-128 23586463 PMC3637064

[B18] Chever O. Lee C. Y. Rouach N. (2014). Astroglial connexin43 hemichannels tune basal excitatory synaptic transmission. *J. Neurosci.* 34 11228–11232. 10.1523/JNEUROSCI.0015-14.2014 25143604 PMC6615508

[B19] Cisterna B. A. Cardozo C. Saez J. C. (2014). Neuronal involvement in muscular atrophy. *Front. Cell Neurosci.* 8:405. 10.3389/fncel.2014.00405 25540609 PMC4261799

[B20] Cisterna B. A. Vargas A. A. Puebla C. Fernández P. Escamilla R. Lagos C. F. (2020). Active acetylcholine receptors prevent the atrophy of skeletal muscles and favor reinnervation. *Nat. Commun.* 11:1073. 10.1038/s41467-019-14063-8 32103010 PMC7044284

[B21] Contreras J. E. Sáez J. Bukauskas F. Bennett M. (2003). Gating and regulation of connexin 43 (Cx43) hemichannels. *Proc. Natl. Acad. Sci. U S A.* 100 11388–11393. 10.1073/pnas.1434298100 13130072 PMC208767

[B22] Courtine G. Song B. Roy R. R. Zhong H. Herrmann J. Ao Y. (2008). Recovery of supraspinal control of stepping via indirect propriospinal relay connections after spinal cord injury. *Nat. Med.* 14 69–74. 10.1038/nm1682 18157143 PMC2916740

[B23] Cummings B. J. Engesser-Cesar C. Cadena G. Anderson A. (2007). Adaptation of a ladder beam walking task to assess locomotor recovery in mice following spinal cord injury. *Behav. Brain Res.* 177 232–241. 10.1016/j.bbr.2006.11.042 17197044 PMC1892162

[B24] de Souza P. da Silva R. da Silva L. Steimbach V. Moreno K. Gasparotto Junior A. (2022). Boldine, an alkaloid from peumus boldus molina, induces endothelium-dependent vasodilation in the perfused rat kidney: involvement of nitric oxide and small-conductance Ca(2+)-activated K(+) channel. *Evid. Based Complement Alternat. Med.* 2022:4560607. 10.1155/2022/4560607 35222671 PMC8865971

[B25] Di Virgilio F. Sanz J. Chiozzi P. Falzoni S. (1999). The P2Z/P2X7 receptor of microglial cells: a novel immunomodulatory receptor. *Prog Brain Res.* 120 355–368. 10.1016/S0079-6123(08)63569-4 10551011

[B26] Estrada M. Uhlen P. Ehrlich B. E. (2006). Ca2+ oscillations induced by testosterone enhance neurite outgrowth. *J. Cell Sci.* 119 733–743. 10.1242/jcs.02775 16449326

[B27] Evans W. H. De Vuyst E. Leybaert L. (2006). The gap junction cellular internet: connexin hemichannels enter the signalling limelight. *Biochem. J.* 397 1–14. 10.1042/BJ20060175 16761954 PMC1479757

[B28] Fabbiani G. Reali C. Valentín-Kahan A. Rehermann M. Fagetti J. Falco M. (2020). Connexin signaling is involved in the reactivation of a latent stem cell niche after spinal cord injury. *J. Neurosci.* 40 2246–2258. 10.1523/JNEUROSCI.2056-19.2020 32001613 PMC7083287

[B29] Filipp M. E. Travis B. Henry S. Idzikowski E. Magnuson S. Loh M. (2019). Differences in neuroplasticity after spinal cord injury in varying animal models and humans. *Neural Regen. Res.* 14 7–19. 10.4103/1673-5374.243694 30531063 PMC6263009

[B30] Fornaro M. Giovannelli A. Foggetti A. Muratori L. Geuna S. Novajra G. (2020). Role of neurotrophic factors in enhancing linear axonal growth of ganglionic sensory neurons in vitro. *Neural Regen. Res.* 15 1732–1739. 10.4103/1673-5374.276338 32209780 PMC7437584

[B31] Garcia-Rodriguez C. Bravo-Tobar I. Duarte Y. Barrio L. Sáez J. (2022). Contribution of non-selective membrane channels and receptors in epilepsy. *Pharmacol. Ther.* 231:107980. 10.1016/j.pharmthera.2021.107980 34481811

[B32] García-Rodríguez C. Bravo-Tobar I. Duarte Y. Barrio L. Sáez J. (2022). Contribution of non-selective membrane channels and receptors in epilepsy. *Pharmacol. Therapeutics* 231:107980. 10.1016/j.pharmthera.2021.107980 34481811

[B33] Gene Ontology C. (2021). The gene ontology resource: enriching a gold mine. *Nucleic Acids Res.* 49 D325–D334. 33290552 10.1093/nar/gkaa1113 PMC7779012

[B34] Giaume C. Naus C. Sáez J. Leybaert L. (2021). Glial connexins and pannexins in the healthy and diseased brain. *Physiol. Rev.* 101 93–145. 10.1152/physrev.00043.2018 32326824

[B35] Hayakawa K. Okazaki R. Ishii K. Ueno T. Izawa N. Tanaka Y. (2012). Phosphorylated neurofilament subunit NF-H as a biomarker for evaluating the severity of spinal cord injury patients, a pilot study. *Spinal Cord* 50 493–496. 10.1038/sc.2011.184 22270191

[B36] Huang C. Han X. Li X. Lam E. Peng W. Lou N. (2012). Critical role of connexin 43 in secondary expansion of traumatic spinal cord injury. *J. Neurosci.* 32 3333–3338. 10.1523/JNEUROSCI.1216-11.2012 22399755 PMC3569730

[B37] Kerstetter A. E. Miller R. H. (2012). “Isolation and culture of spinal cord astrocytes,” in *Astrocytes: Methods and Protocols*, ed. Milner R. (Totowa, NJ: Humana Press). 10.1007/978-1-61779-452-0_7 PMC3568947 22144302

[B38] Koshi T. Ohtori S. Inoue G. Ito T. Yamashita M. Yamauchi K. (2010). Lumbar posterolateral fusion inhibits sensory nerve ingrowth into punctured lumbar intervertebral discs and upregulation of CGRP immunoreactive DRG neuron innervating punctured discs in rats. *Eur. Spine J.* 19 593–600. 10.1007/s00586-009-1237-9 20012755 PMC2899833

[B39] Koshimizu T. Koshimizu M. Stojilkovic S. S. (1999). Contributions of the C-terminal domain to the control of P2X receptor desensitization. *J. Biol. Chem.* 274 37651–37657. 10.1074/jbc.274.53.37651 10608821

[B40] Lee I. H. Lindqvist E. Kiehn O. Widenfalk J. Olson L. (2005). Glial and neuronal connexin expression patterns in the rat spinal cord during development and following injury. *J. Comp. Neurol.* 489 1–10. 10.1002/cne.20567 15977163

[B41] Mao Y. Nguyen T. Tonkin R. Lees J. Warren C. O’Carroll S. (2017). Characterisation of Peptide5 systemic administration for treating traumatic spinal cord injured rats. *Exp. Brain Res.* 235 3033–3048. 10.1007/s00221-017-5023-3 28725925

[B42] Mariottini C. Munari L. Gunzel E. Seco J. Tzavaras N. Hansen J. (2019). Wilm’s tumor 1 promotes memory flexibility. *Nat. Commun.* 10:3756. 10.1038/s41467-019-11781-x 31434897 PMC6704057

[B43] Miller A. D. Westmoreland S. Evangelous N. Graham A. Sledge J. Nesathurai S. (2012). Acute traumatic spinal cord injury induces glial activation in the cynomolgus macaque (*Macaca fascicularis*). *J. Med. Primatol.* 41 202–209. 10.1111/j.1600-0684.2012.00542.x 22620270 PMC3367394

[B44] Munoz M. F. Griffith T. N. Contreras J. E. (2021). Mechanisms of ATP release in pain: role of pannexin and connexin channels. *Purinergic Signal.* 17 549–561. 10.1007/s11302-021-09822-6 34792743 PMC8677853

[B45] Nagy J. I. Ionescu A. Lynn B. Rash J. (2003). Coupling of astrocyte connexins Cx26, Cx30, Cx43 to oligodendrocyte Cx29, Cx32, Cx47: implications from normal and connexin32 knockout mice. *Glia* 44 205–218. 10.1002/glia.10278 14603462 PMC1852517

[B46] O’Carroll S. J. Gorrie C. Velamoor S. Green C. Nicholson L. (2013). Connexin43 mimetic peptide is neuroprotective and improves function following spinal cord injury. *Neurosci. Res.* 75 256–267. 10.1016/j.neures.2013.01.004 23403365

[B47] Okada S. Hara M. Kobayakawa K. Matsumoto Y. Nakashima Y. (2018). Astrocyte reactivity and astrogliosis after spinal cord injury. *Neurosci. Res.* 126 39–43. 10.1016/j.neures.2017.10.004 29054466

[B48] Oliveira A. L. Thams S. Lidman O. Piehl F. Hökfelt T. Kärre K. (2004). A role for MHC class I molecules in synaptic plasticity and regeneration of neurons after axotomy. *Proc. Natl. Acad. Sci. U S A.* 101 17843–17848. 10.1073/pnas.0408154101 15591351 PMC539738

[B49] O’Shea T. M. Burda J. E. Sofroniew M. V. (2017). Cell biology of spinal cord injury and repair. *J. Clin. Invest.* 127 3259–3270. 10.1172/JCI90608 28737515 PMC5669582

[B50] Peng S. Wuu J. Mufson E. Fahnestock M. (2005). Precursor form of brain-derived neurotrophic factor and mature brain-derived neurotrophic factor are decreased in the pre-clinical stages of Alzheimer’s disease. *J. Neurochem.* 93 1412–1421. 10.1111/j.1471-4159.2005.03135.x 15935057

[B51] Peng W. Cotrina M. Han X. Yu H. Bekar L. Blum L. (2009). Systemic administration of an antagonist of the ATP-sensitive receptor P2X7 improves recovery after spinal cord injury. *Proc. Natl. Acad. Sci. U S A.* 106 12489–12493. 10.1073/pnas.0902531106 19666625 PMC2718350

[B52] Potter L. A. Toro C. Harlow L. Lavin K. Cardozo C. Wende A. (2023). Assessing the impact of boldine on the gastrocnemius using multiomic profiling at 7 and 28 days post-complete spinal cord injury in young male mice. *Physiol. Genomics* Online ahead of print. 10.1152/physiolgenomics.00129.2022 37125768 PMC10292965

[B53] Rash J. E. Yasumura T. Davidson K. Furman C. Dudek F. Nagy J. (2001). Identification of cells expressing Cx43, Cx30, Cx26, Cx32 and Cx36 in gap junctions of rat brain and spinal cord. *Cell Commun. Adhes* 8 315–320. 10.3109/15419060109080745 12064610 PMC1805789

[B54] Retamal M. A. Froger N. Palacios-Prado N. Ezan P. Sáez P. Sáez J. (2007). Cx43 hemichannels and gap junction channels in astrocytes are regulated oppositely by proinflammatory cytokines released from activated microglia. *J. Neurosci.* 27 13781–13792. 10.1523/JNEUROSCI.2042-07.2007 18077690 PMC6673621

[B55] Russ D. E. Cross R. Li L. Koch S. Matson K. Yadav A. (2021). A harmonized atlas of mouse spinal cord cell types and their spatial organization. *Nat. Commun.* 12:5722. 10.1038/s41467-021-25125-1 34588430 PMC8481483

[B56] Ryden M. Hempstead B. Ibanez C. F. (1997). Differential modulation of neuron survival during development by nerve growth factor binding to the p75 neurotrophin receptor. *J. Biol. Chem.* 272 16322–16328. 10.1074/jbc.272.26.16322 9195937

[B57] Sáez J. C. Cisterna B. Vargas A. Cardozo C. (2015). Regulation of pannexin and connexin channels and their functional role in skeletal muscles. *Cell Mol. Life Sci.* 72 2929–2935. 10.1007/s00018-015-1968-1 26084874 PMC11113819

[B58] Schalper K. A. Sánchez H. Lee S. Altenberg G. Nathanson M. Sáez J. (2010). Connexin 43 hemichannels mediate the Ca2+ influx induced by extracellular alkalinization. *Am. J. Physiol. Cell Physiol.* 299 C1504–C1515. 10.1152/ajpcell.00015.2010 20881238 PMC3774097

[B59] Scheff S. W. Rabchevsky A. Fugaccia I. Main J. Lumpp J. (2003). Experimental modeling of spinal cord injury: characterization of a force-defined injury device. *J. Neurotrauma* 20 179–193. 10.1089/08977150360547099 12675971

[B60] Shintani-Ishida K. Uemura K. Yoshida K. (2007). Hemichannels in cardiomyocytes open transiently during ischemia and contribute to reperfusion injury following brief ischemia. *Am. J. Physiol. Heart Circ. Physiol.* 293 H1714–H1720. 10.1152/ajpheart.00022.2007 17557925

[B61] Sluyter R. Stokes L. (2011). Significance of P2X7 receptor variants to human health and disease. *Recent Pat DNA Gene Seq.* 5 41–54. 10.2174/187221511794839219 21303345

[B62] Spiller K. J. Khan T. Dominique M. Restrepo C. Cotton-Samuel D. Levitan M. (2019). Reduction of matrix metalloproteinase 9 (MMP-9) protects motor neurons from TDP-43-triggered death in rNLS8 mice. *Neurobiol. Dis.* 124 133–140. 10.1016/j.nbd.2018.11.013 30458231 PMC7053168

[B63] Spray D. C. Hanani M. (2019). Gap junctions, pannexins and pain. *Neurosci. Lett.* 695 46–52. 10.1016/j.neulet.2017.06.035 28647288 PMC6005766

[B64] Streijger F. Lee J. Manouchehri N. Melnyk A. Chak J. Tigchelaar S. (2016). Responses of the acutely injured spinal cord to vibration that simulates transport in helicopters or mine-resistant ambush-protected vehicles. *J. Neurotrauma* 33 2217–2226. 10.1089/neu.2016.4456 27214588

[B65] Suadicani S. O. Brosnan C. F. Scemes E. (2006). P2X7 receptors mediate ATP release and amplification of astrocytic intercellular Ca2+ signaling. *J. Neurosci.* 26 1378–1385. 10.1523/JNEUROSCI.3902-05.2006 16452661 PMC2586295

[B66] Theriault E. Frankenstein U. Hertzberg E. Nagy J. (1997). Connexin43 and astrocytic gap junctions in the rat spinal cord after acute compression injury. *J. Comp. Neurol.* 382 199–214. 10.1002/(SICI)1096-9861(19970602)382:2<199::AID-CNE5>3.0.CO;2-Z 9183689

[B67] Toro C. A. Hansen J. Siddiq M. Johnson K. Zhao W. Azulai D. (2021). The human ApoE4 variant reduces functional recovery and neuronal sprouting after incomplete spinal cord injury in male mice. *Front. Cell Neurosci.* 15:626192. 10.3389/fncel.2021.626192 33679326 PMC7930340

[B68] Toro C. A. Wright H. Aylwin C. Ojeda S. Lomniczi A. (2018). Trithorax dependent changes in chromatin landscape at enhancer and promoter regions drive female puberty. *Nat. Commun.* 9:57. 10.1038/s41467-017-02512-1 29302059 PMC5754362

[B69] Turney S. G. Ahmed M. Chandrasekar I. Wysolmerski R. Goeckeler Z. Rioux R. (2016). Nerve growth factor stimulates axon outgrowth through negative regulation of growth cone actomyosin restraint of microtubule advance. *Mol. Biol. Cell* 27 500–517. 10.1091/mbc.e15-09-0636 26631553 PMC4751601

[B70] Walstab J. Wohlfarth C. Hovius R. Schmitteckert S. Röth R. Lasitschka F. (2014). Natural compounds boldine and menthol are antagonists of human 5-HT3 receptors: implications for treating gastrointestinal disorders. *Neurogastroenterol. Motil.* 26 810–820. 10.1111/nmo.12334 24708203

[B71] Wang F. Flanagan J. Su N. Wang L. Bui S. Nielson A. (2012). RNAscope: a novel in situ RNA analysis platform for formalin-fixed, paraffin-embedded tissues. *J. Mol. Diagn.* 14 22–29. 10.1016/j.jmoldx.2011.08.002 22166544 PMC3338343

[B72] Wang X. Arcuino G. Takano T. Lin J. Peng W. Wan P. (2004). P2X7 receptor inhibition improves recovery after spinal cord injury. *Nat. Med.* 10 821–827. 10.1038/nm1082 15258577

[B73] Xuan F. L. Chithanathan K. Lilleväli K. Yuan X. Tian L. (2019). Differences of microglia in the brain and the spinal cord. *Front. Cell Neurosci.* 13:504. 10.3389/fncel.2019.00504 31803021 PMC6868492

[B74] Yi C. Ezan P. Fernández P. Schmitt J. Sáez J. Giaume C. (2017). Inhibition of glial hemichannels by boldine treatment reduces neuronal suffering in a murine model of Alzheimer’s disease. *Glia* 65 1607–1625. 10.1002/glia.23182 28703353

[B75] Zhang C. Yan Z. Maknojia A. Riquelme M. Gu S. Booher G. (2021). Inhibition of astrocyte hemichannel improves recovery from spinal cord injury. *JCI Insight* 6:e134611. 10.1172/jci.insight.134611 33682795 PMC8021110

[B76] Zhong J. Carrozza D. Williams K. Pritchett D. Molinoff P. (1995). Expression of mRNAs encoding subunits of the NMDA receptor in developing rat brain. *J. Neurochem.* 64 531–539. 10.1046/j.1471-4159.1995.64020531.x 7830045

