# Supplementary figures and images for: Boldine modulates glial transcription and functional recovery in a murine model of contusion spinal cord injury
Source: Front Cell Neurosci. 2023 Jun 21;17:1163436. doi: 10.3389/fncel.2023.1163436 (PMC10321410; doi:10.3389/fncel.2023.1163436)

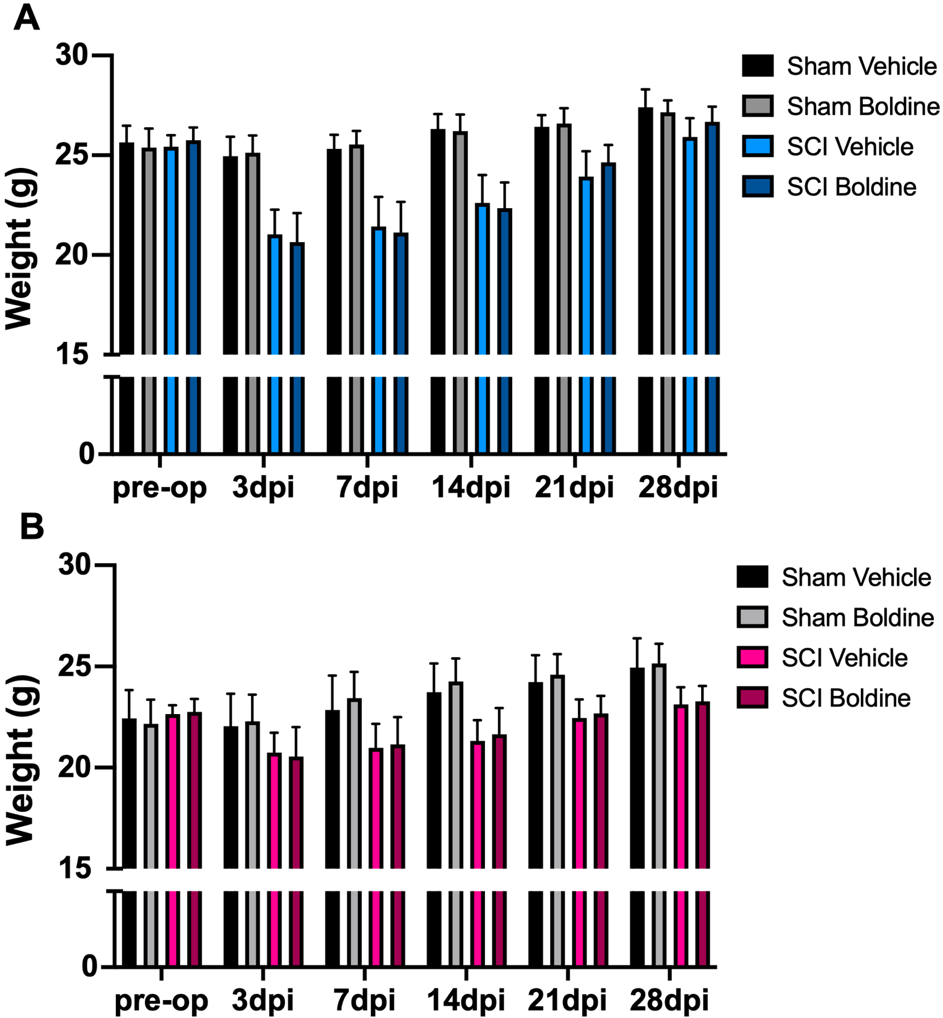

Supplement: Supplementary Figure 1 — Body weights were determined on the day of surgery prior to making an incision (pre-op) and at the specified times shown on the X-axis for male (A) and female (B) mice. Data are shown as mean values ± SEM. [file Image_1.TIFF]

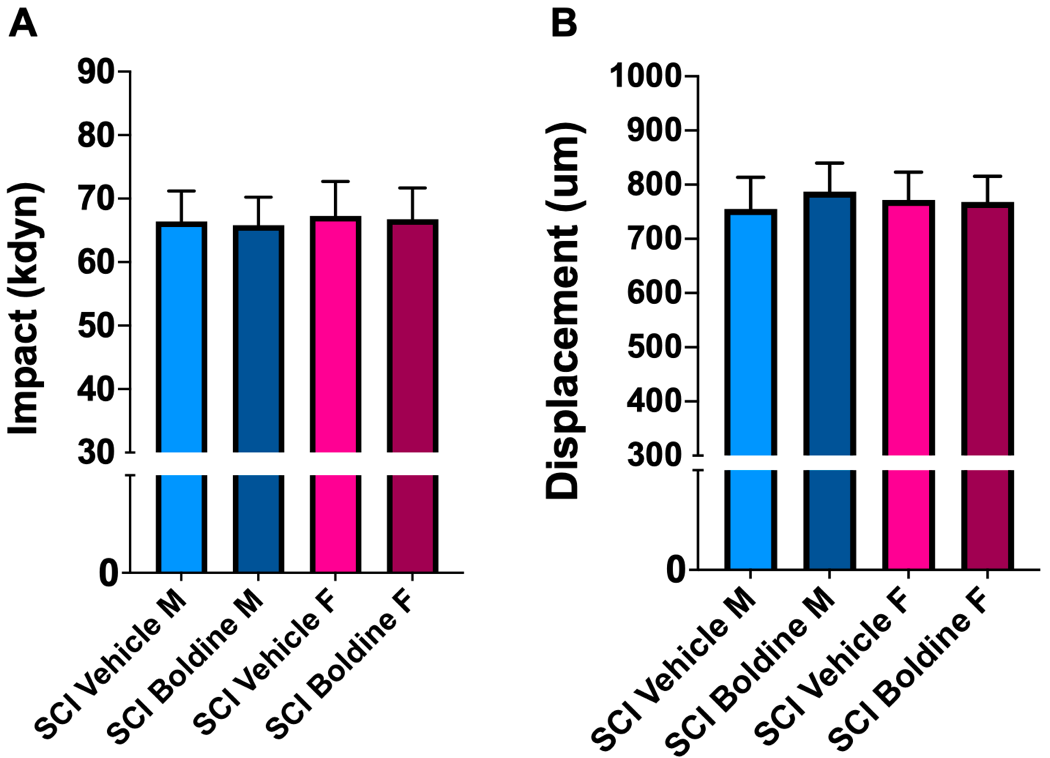

Supplement: Supplementary Figure 2 — Actual impact force (kdyne) (A) and spinal cord displacement during impact (μm) (B) are shown. Data are shown as mean ± SEM. [file Image_2.TIFF]

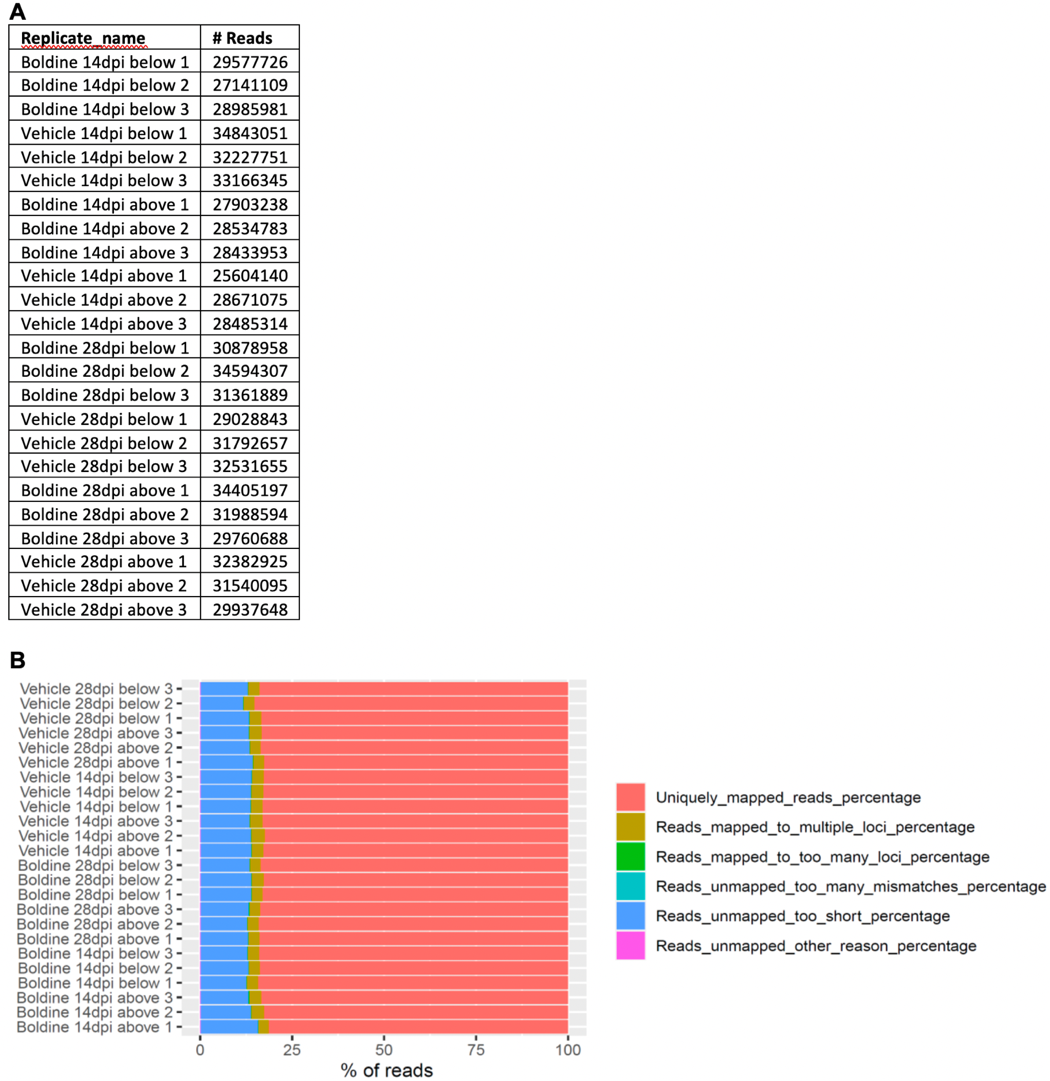

Supplement: Supplementary Figure 3 — Transcriptomic profiling by RNA sequencing. “Star” was used for read alignment to the mouse reference genome. (A) Read counts and (# Reads) (B) alignment efficiencies are shown for each sample. [file Image_3.TIFF]

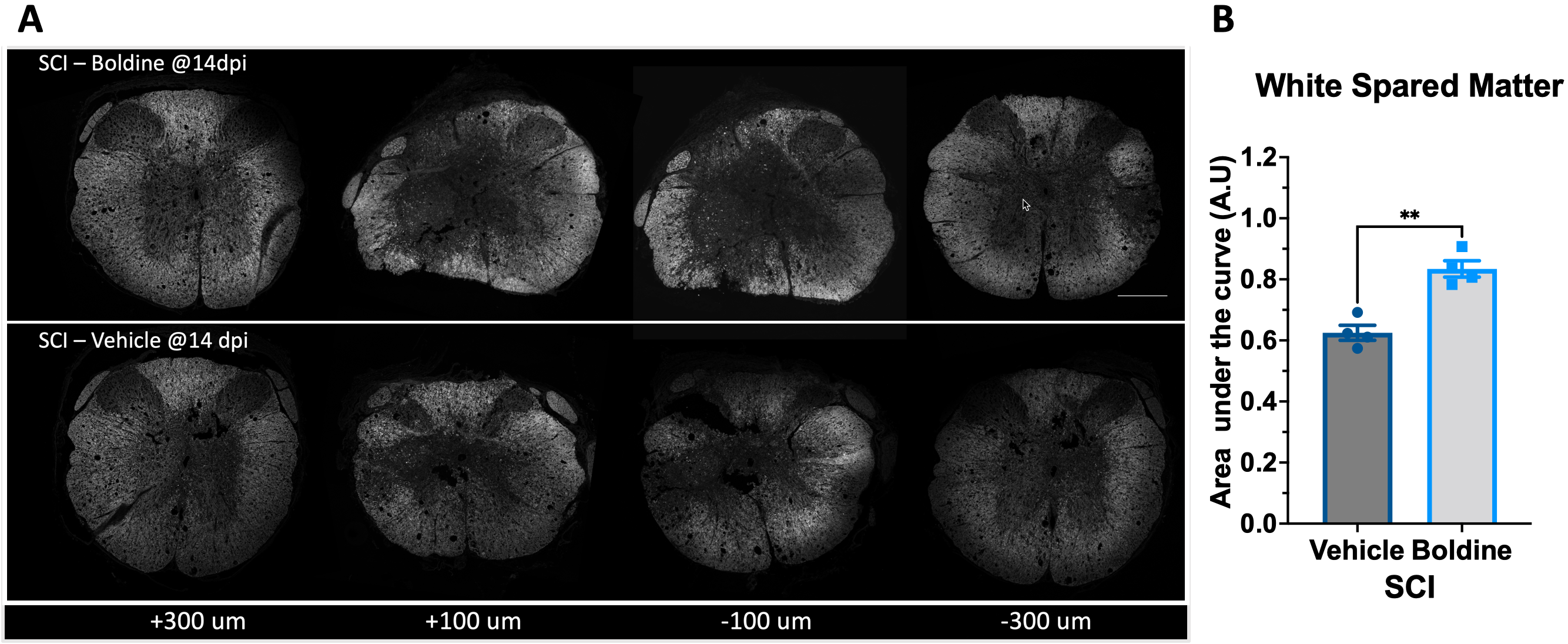

Supplement: Supplementary Figure 4 — Boldine promotes sparing of white matter at 14 days post SCI. Perfusion-fixed spinal cords of boldine and vehicle treated SCI animals were cryo-sectioned. Transverse sections were collected at 100 and 300 μm rostral and caudal from the injury the epicenter and stained with FluoroMyelin. Panel (A) shows representative images at +300, +100, −100 and −300 μm for each group. (B) White matter sparing was compared between boldine and vehicle-treated SCI groups. Bar plots are presented as mean ± SEM. Statistical analysis was performed using unpaired t-test. **p < 0.01 N = 3 per group. Scale bar is 500 μm. [file Image_4.TIFF]

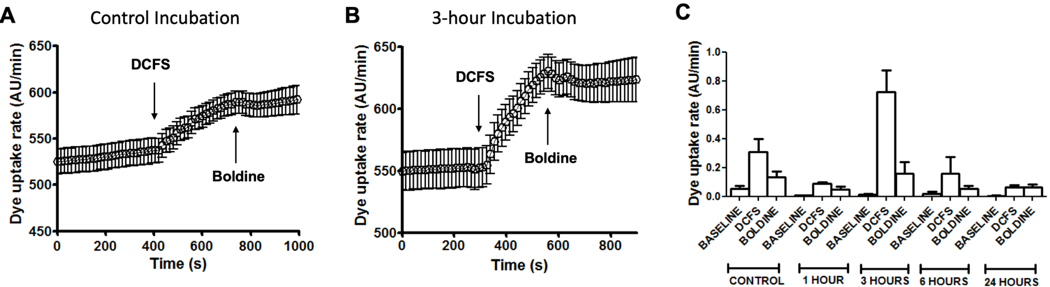

Supplement: Supplementary Figure 5 — Boldine blocks hemichannels expressed by activated spinal cord astrocytes. Spinal cord astrocytes were cultured under control conditions or treated with 10 ng/ml TNF-α plus 10 ng/ml IL-1β 1 at different time periods and dye uptake measured as fluorescence intensity in arbitrary units (AU) was evaluated under basal conditions, after exposure to extracellular divalent cation-free solution (DCFS) followed by the application of 50 μM boldine at the times denoted by the arrows in (A) and (B). Curve of fluorescence intensity over time for dye uptake rate was calculated for each condition (C). Each value represents the mean ± SEM of three independent experiments. A total of 30 cells were recorded per experiment. [file Image_5.TIFF]

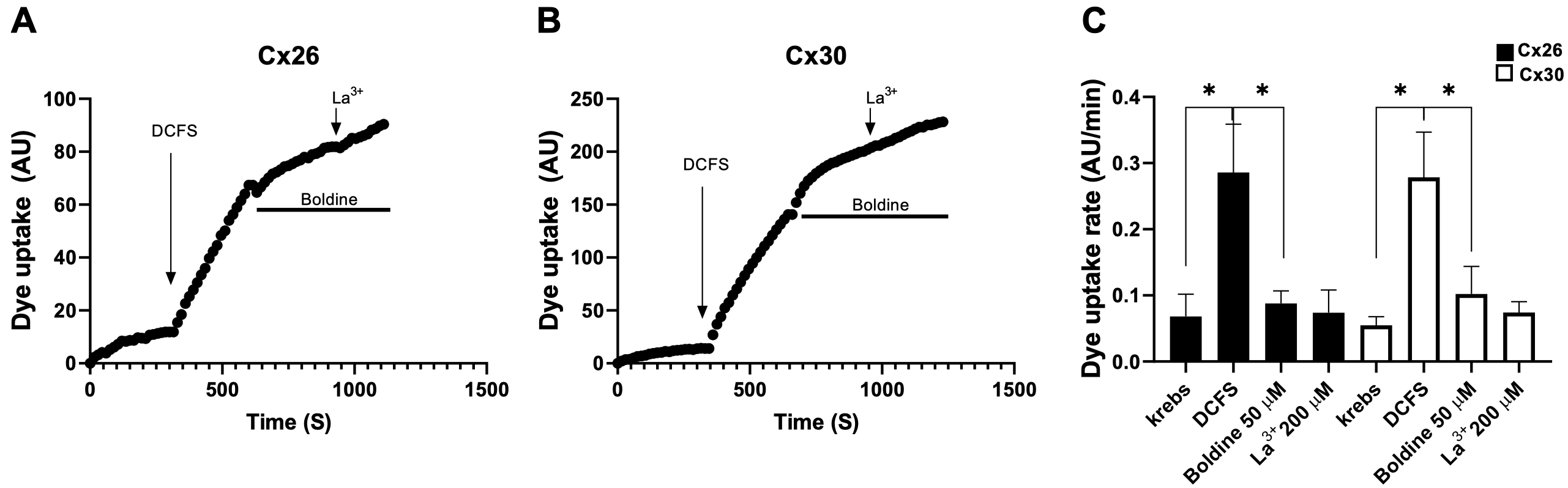

Supplement: Supplementary Figure 6 — Boldine blocks Cx26 and Cx30 hemichannels. (A,B) Cx HC activity was assessed by DAPI uptake measure in time-lapse in Krebs solution, in DCFS to increase the open probability of hemichannels, and in DCFS plus 50 μM boldine. (C) DAPI uptake rate in HeLa Cx26 y HeLa Cx30, the application of DCFS increases the slope of the dye uptake curve and the application boldine in DCFS drastically reduced the dye uptake. N = 4, 30 cells were recorded for each experiment, values are presented as the mean ± SEM. *p < 0.05. Tukey’s multiple comparisons test. [file Image_6.TIFF]

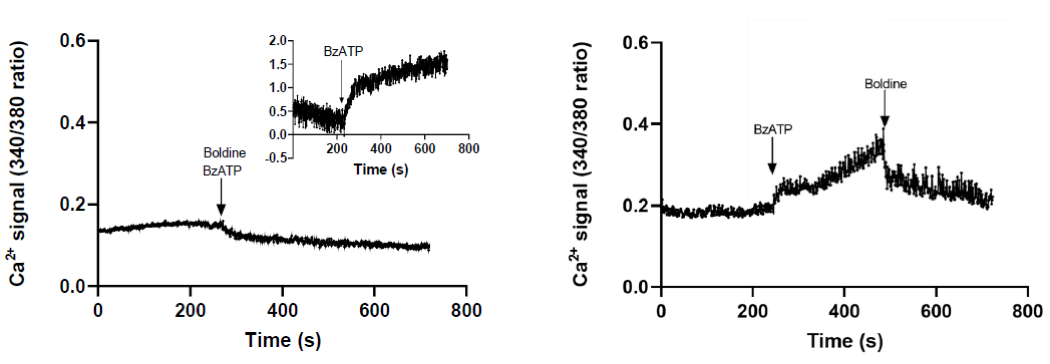

Supplement: Supplementary Figure 7 — Boldine blocks P2X7R. The activity of P2X7R was evaluated in HeLa cells transiently transfected with mouse P2X7R -EGFP. Cells were loaded with Fura-2 and intracellular calcium signal was evaluated. Upon treatment with 100 μM benzoyl ATP (inset in the left panel) a calcium signal increase was evident. In some experiments 50 μM boldine (arrows) was added simultaneously with BzATP (left panel) or after cells were treated with BzATP (right panel). Each plotted point corresponds to the mean value of 20 cells of a representative experiment out of four independent experiments. [file Image_7.TIFF]

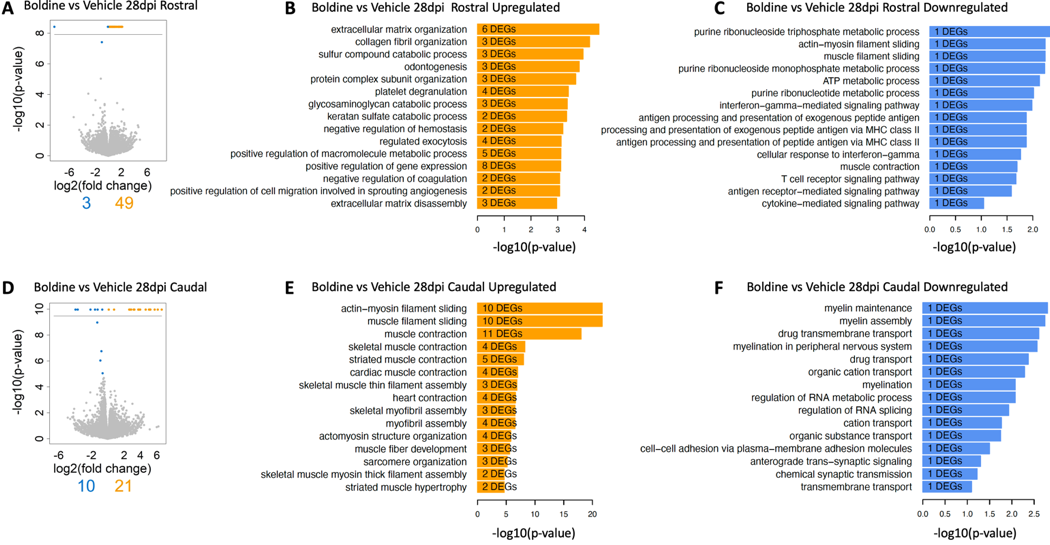

Supplement: Supplementary Figure 8 — Results of analysis by bulk-RNA sequencing of total RNA isolated from spinal cord segments rostral and caudal from the lesion site at 28 dpi. Spinal cord segments collected at 28 dpi from mice with contusion SCI treated with boldine or vehicle were subjected to bulk-RNA sequencing. Boldine-induced DEGs (FDR 10%) were identified between boldine and vehicle treated SCI animals either rostral (A–C) or caudal (D–F) from the injury epicenter. (A,D) Blue and orange dots indicate significantly up- or downregulated genes, respectively. DEGs predicted with a p-value of 0 and consequently −log10(p-value) of infinity are visualized above the horizontal line. (B,C,E,F) Up-regulated and downregulated genes for a particular condition were subjected to pathway enrichment analysis using Gene Ontology Biological Process and Fisher’s Exact test, followed by ranking of the predicted pathways by significance. The top 15 ranked pathways are shown for all lists that describe differences between treatments rostral or caudal from the lesion site: (B) Boldine (vs. vehicle) rostral from the lesion upregulated, (C) Boldine (vs. vehicle) rostral from the lesion downregulated, (E) Boldine (vs. vehicle) caudal from the lesion upregulated, and (F) Boldine (vs. vehicle) caudal from the lesion downregulated. Numbers of DEGs observed in each particular pathway are shown within the bar for that pathway. Procedures were as described in the Section “Materials and methods” and the legend to Figure 5. [file Image_8.TIFF]

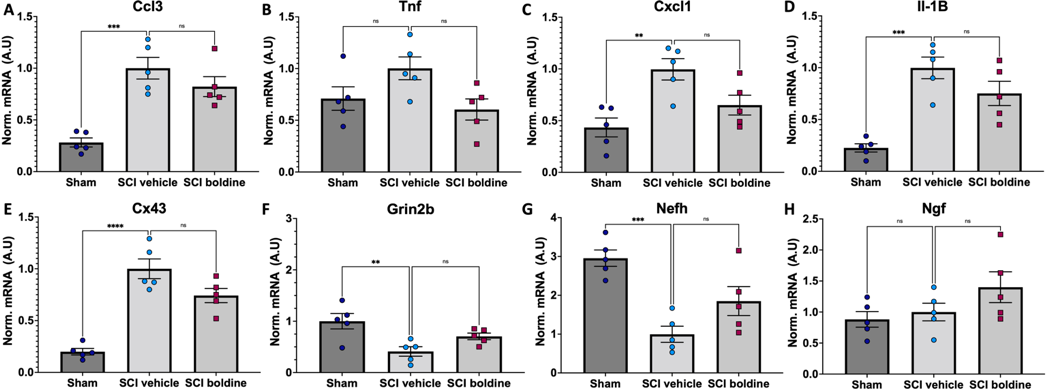

Supplement: Supplementary Figure 9 — Additional RT-qPCR Results. RT-qPCR was performed using total RNA isolated from 4 mm spinal cord segments spanning T7 to T11 from sham and SCI mice at 14 dpi. Levels of (A) Ccl3; F(2, 8) = 15.62, (B) Tnf; F(2, 8) = 4.54, (C) Cxcl1 F(2, 8) = 10.23, (D) Il-1b; F(2, 8) = 13.88, (E) Cx43; F(2, 8) = 36.82, (F) Grin2b; F(2, 8) = 5.727, (G) Nefh; F(2, 8) = 13.37, and (H) Ngf; F(2, 8) = 2.16 were detected for laminectomy-only (Sham), and for SCI animals treated with vehicle (SCI vehicle) or boldine (SCI boldine). Data are expressed as arbitrary units (AU) after normalizing to SCI vehicle samples. Bar plots show mean ± SEM. Statistical analysis was performed by one-way ANOVA followed by Tukey’s multiple comparisons test *p < 0.05; **p < 0.005; ***p < 0.001; ****p < 0.0001. N = 5. [file Image_9.TIFF]
